# Establishment of an ulcerative colitis model using colon organoids derived from human induced pluripotent stem cells

**DOI:** 10.1016/j.isci.2024.111049

**Published:** 2024-09-26

**Authors:** Fuki Yokoi, Sayaka Deguchi, Yukio Watanabe, Kazuo Takayama

**Affiliations:** 1 Center for iPS Cell Research and Application (CiRA), Kyoto University, Kyoto 606-8507, Japan; 2 Department of Human Health Sciences, Graduate School of Medicine, Kyoto University, Kyoto 606-8501, Japan; 3 AMED-CREST, Japan Agency for Medical Research and Development (AMED), Tokyo 100-0004, Japan

**Keywords:** Molecular biology, Cell biology, Stem cells research, Transcriptomics

## Abstract

The etiology of inflammatory bowel disease (IBD) is complex, with much room for a greater understanding and development of improved therapies. Therefore, establishing a reliable IBD model is crucial for future advancements. In this study, human induced pluripotent stem (iPS) cell-derived colon organoids (hiPSC-COs) were treated with a combination of tumor necrosis factor alpha (TNF-α), interferon-gamma (IFN-γ), and interleukin (IL)-1β (3 cytokines [3CK]), known to be elevated in the serum of IBD patients. Inflammatory responses in stromal cells and damage to intestinal epithelial cells were observed in the 3CK-treated hiPSC-COs. Comparison of molecular signatures of 3CK-treated hiPSC-COs with those of ulcerative colitis (UC) patient’s colon revealed that 3CK-treated hiPSC-COs resemble UC patient’s colon. Furthermore, the elevated production of inflammatory cytokines observed in 3CK-treated hiPSC-COs was attenuated by treatment with tofacitinib. Our UC model will be an essential tool to understand its pathologic mechanisms and identify effective therapeutic approaches.

## Highlights

• An ulcerative colitis (UC) model was established using human iPS cell-derived colon organoids
• Epithelial cells were damaged and stromal cells produced inflammatory cytokines
• Our UC model effectively emulates the pathophysiology of UC colon
• The therapeutic effects of tofacitinib could be evaluated using our UC model

## Graphical abstract

**Figure:**
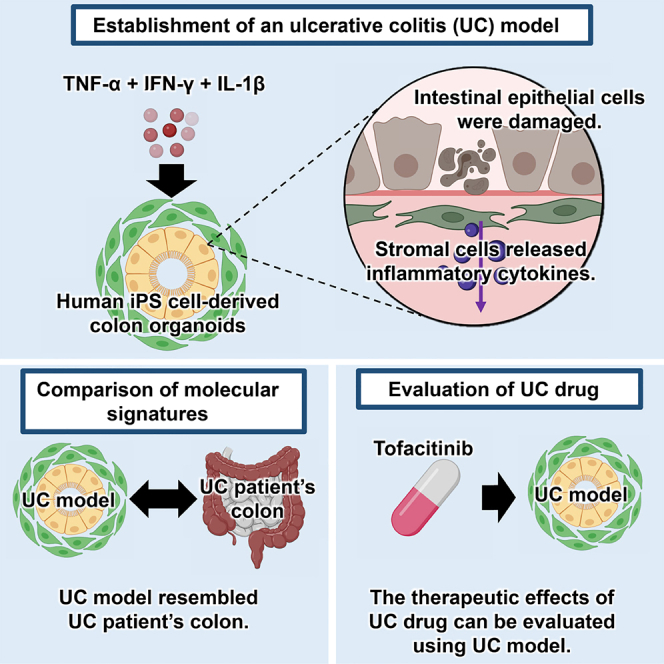

## Introduction

Chronic inflammation is observed in the intestines in patients with inflammatory bowel disease (IBD). Gastrointestinal symptoms, such as diarrhea and abdominal pain, are frequently observed in patients with IBD.^1^ Steroids, 5-aminosalicylic acid, and Janus kinase (JAK) inhibitors are currently used to treat IBD. However, these drugs do not cure the disease, and remission rates of IBD are below 60%.^2^ Therefore, there is room for further development of IBD treatments. Although IBD is considered to be caused by a variety of factors, including genetic predisposition, environmental triggers, and dysbiosis, its causes are not fully understood. In the intestinal tract of IBD patients, immune cells secrete excessive inflammatory cytokines, such as tumor necrosis factor alpha (TNF-α), interferon-gamma (IFN-γ), and interleukin (IL)-1β.^3^ These inflammatory cytokines are one of the causes of enteritis in IBD patients and are thus a critical factor in disrupting colon homeostasis.

The human colon is composed of a variety of cells, including colonic epithelial cells and stromal cells. Colonic epithelial cells are responsible for the major functions of the colon: the absorption of water and minerals and the secretion of mucus. Under the colon epithelial cell layer, stromal cells function to maintain colon homeostasis.^4^ In enteritis, the homeostasis of colonic epithelial and stromal cells is disrupted. For example, in the colon of IBD patients, the expression of metabolic-related genes in colonic epithelial cells is altered while the percentage of fibroblasts expressing inflammatory cytokines is increased.^5^ Therefore, to accurately reproduce the pathophysiology of IBD, it is necessary to construct a colon model that includes both colonic epithelial and stromal cells.

Many researchers have attempted to reproduce IBD pathophysiology using Caco-2 cells^6^ or colon organoids^7^ derived from colon biopsies of patients with ulcerative colitis (UC). Although Caco-2 cells, derived from human colon cancer, are commonly used, they differ substantially from normal colonic epithelial cells. While colon organoids established from colon biopsies are also widely used in IBD research, their main constituent is colonic epithelial cells, with few stromal cells to accurately imitate enteritis.^8^ Therefore, it is necessary to establish a colon model containing colonic epithelial and stromal cells and use them to recapitulate the pathophysiology of IBD.

Human induced pluripotent stem cells (hiPSCs) can differentiate into definitive endoderm ^9^ and mesoderm ^10^ cells, thus making it possible to construct a colon organoid model containing colonic epithelial and stromal cells. With such hiPSC-derived colon organoids (hiPSC-COs), it may be possible to examine IBD pathophysiology with far more details than existing *in vitro* colon models. In this study, we developed a method to simultaneously differentiate colonic epithelial and stromal cells from hiPSCs. Next, we attempted to reproduce the pathophysiology of IBD by treating these colon organoids with IBD-related inflammatory cytokines and validated the resulting IBD organoid model by comparing its gene expression profile with active site of colon tissues from IBD patients. Finally, we examined the potential of this IBD model for drug discovery applications by examining the efficacy of tofacitinib, a Food and Drug Administration-approved drug for UC, to reverse IBD pathophysiological changes.

## Results

### Generation of hiPSC-COs containing both colonic epithelial and stromal cells

**Figure 1:**
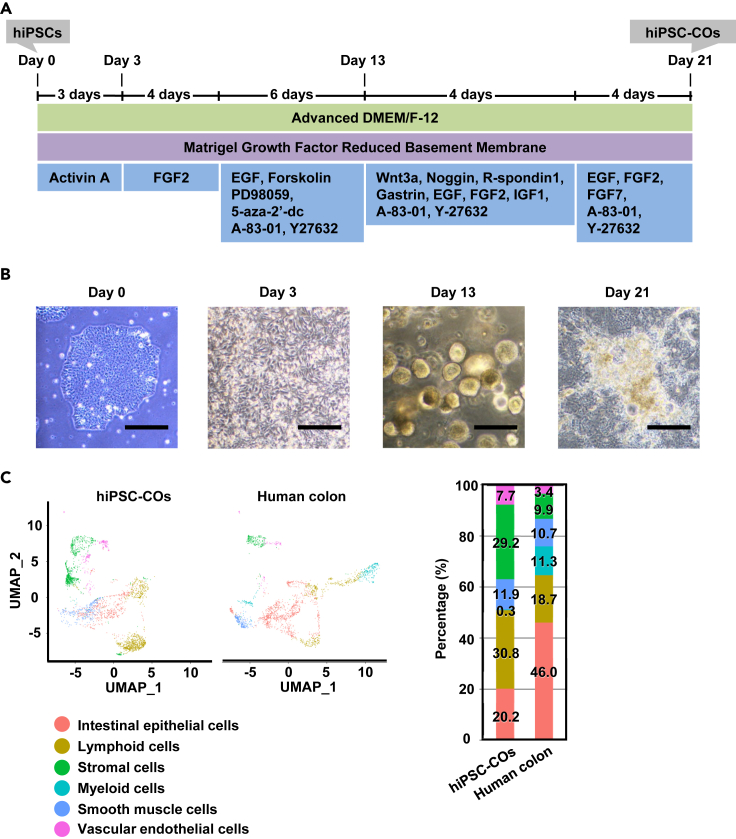
Generation of hiPSC-COs containing both colonic epithelial cells and stromal cells (A) hiPSCs were differentiated into colon organoids. (B) Phase contrast images were obtained during the differentiation of colon organoids from hiPSCs (days 0, 3, 13, and 21 of the differentiation). Scale bars represent 200 μm. (C) The scRNA-seq analysis of hiPSC-COs was performed. UMAP plots of hiPSC-COs and the human colon are shown on the left. Relative proportions of each cluster in hiPSC-COs and the human colon are shown on the right. Gene lists shown in Table S1 were used for cell-type annotation in scRNA-seq analysis. See also Figure S1.

We attempted to generate colon organoids containing not only colonic epithelial cells but also stromal cells from the hiPSC line, 1383D6, which was established from peripheral blood cells of a healthy individual (Figure 1A). As schematically shown in Figure 1A, hiPSCs were differentiated into colon organoids in five steps. Phase contrast images of hiPSC-COs at each stage are shown in Figure 1B. Gene expression of intestinal epithelial cell markers (*villin 1* [*VIL1*] and *sucrase-isomaltase* [*SI*]), goblet cell markers (*trefoil factor 3* [*TFF3*] and *mucin 2* [*MUC2*]), endocrine cell markers (*regenerating family member 4* [*REG4*] and *chromogranin A* [*CHGA*]), and intestinal stem cell marker (*leucine-rich repeat-containing G protein-coupled receptor 5* [*LGR5*]) in hiPSC-COs (day 21 of the differentiation) was increased as compared with that in undifferentiated induced pluripotent stem cells (iPSCs) (day 0 of the differentiation), suggesting that hiPSCs were able to differentiate into colonic epithelial cells (Figure S1A). In addition, colonic epithelial-specific markers (*carbonic anhydrase 1* [*CA1*], *carbonic anhydrase 2* [*CA2*], *carbonic anhydrase 4* [*CA4*], *LY6/PLAUR domain containing 8* [*LYPD8*], *SATB homeobox 2* [*SATB2*], and *solute carrier family 9 member A2* [*SLC9A2*]) were also expressed in hiPSC-COs, suggesting that these organoids have colonic characteristics (Figure S1B).^11^^,^^12^^,^^13^ Furthermore, immunofluorescence showed that hiPSC-COs expressed cytokeratin 20 (CK20), a colonic epithelial cell marker (Figure S1C). The protein level of CK20 in hiPSC-COs was approximately 247-fold higher than that in undifferentiated iPSCs (Figure S1D). Additionally, single-cell RNA sequencing (scRNA-seq) analysis was performed to compare the cell composition of hiPSC-COs with that of the human colon. Using a gene list shown in Table S1, 6 clusters, including intestinal epithelial and stromal cells, were identified in both hiPSC-COs and the human colon (Figure 1C). In general, when differentiating definitive endoderm cells and their derivatives from hiPSCs, a high concentration (100 ng/mL) of activin A is used under serum-free condition.^14^ On the other hand, by using 100 ng/mL activin A in the presence of 1% fetal bovine serum, definitive endoderm and mesoderm cells can be differentiated simultaneously. Therefore, in this study, we successfully produced hiPSC-COs not only with colonic epithelial cells derived from definitive endoderm cells but also with stromal cells derived from mesoderm cells.

### Inflammatory responses can be induced in hiPSC-COs by TNF-α, IFN-γ, and IL-1β treatment

**Figure 2:**
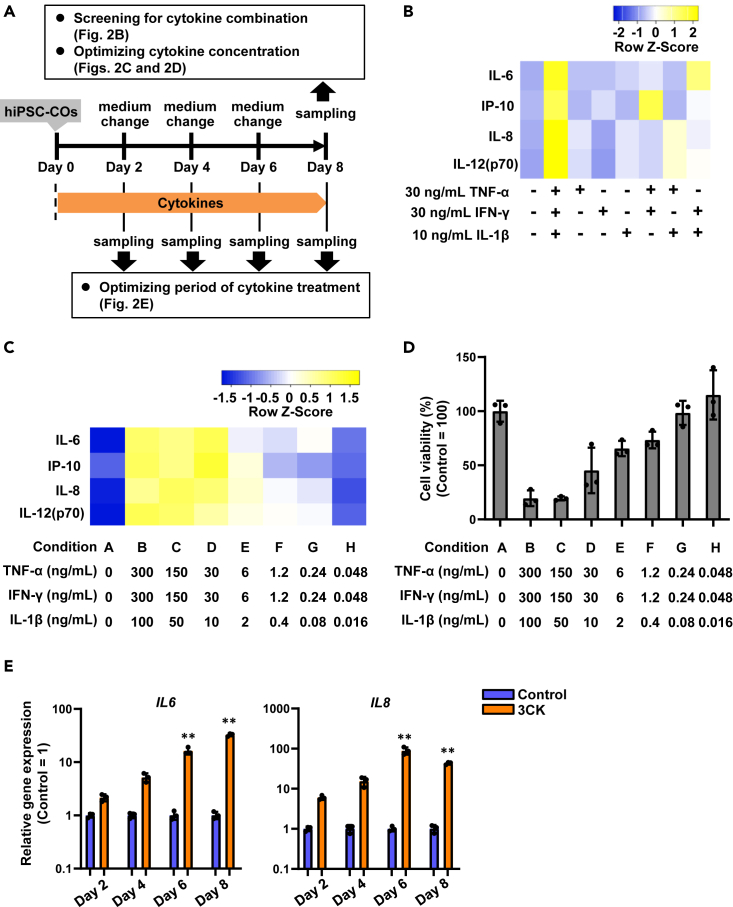
Cytokine screening to induce inflammatory responses in hiPSC-COs (A) hiPSC-COs were treated with IBD-related inflammatory cytokines to induce inflammatory responses. (B) hiPSC-COs were treated with TNF-α (30 ng/mL), IFN-γ (30 ng/mL), or IL-1β (10 ng/mL) for 8 days. Concentrations of inflammatory cytokines secreted from hiPSC-COs were examined by a bead-based multiplex immunoassay (LEGENDplex analysis). Heatmap shows the average concentration of cytokines (IL-6, IP-10 [CXCL10], IL-8, and IL-12(p70)) secreted from TNF-α-, IFN-γ-, or IL-1β-treated hiPSC-COs. (C) hiPSC-COs were treated with different concentrations of TNF-α, IFN-γ, and IL-1β for 8 days. Concentrations of inflammatory cytokines secreted from hiPSC-COs were examined by a bead-based multiplex immunoassay (LEGENDplex analysis). Heatmap shows the average concentration of cytokines (IL-6, IP-10 [CXCL10], IL-8, and IL-12(p70)) secreted from TNF-α-, IFN-γ-, and IL-1β-treated hiPSC-COs. (D) hiPSC-COs were treated with different concentrations of TNF-α, IFN-γ, and IL-1β for 8 days. Cell viability of hiPSC-COs was examined by WST-8 assay, and these values were shown in Table S5. One-way ANOVA followed by the Tukey *post hoc* test. *p* values for all comparisons are shown in Table S6. Data are shown as means ± SD (*n* = 3). (E) hiPSC-COs were treated with TNF-α (30 ng/mL), IFN-γ (30 ng/mL), or IL-1β (10 ng/mL) for 2, 4, 6, and 8 days. Gene expression of inflammatory cytokines (*IL-6* and *IL-8*) in hiPSC-COs was examined by quantitative reverse-transcription PCR (RT-qPCR). Gene expression in control hiPSC-COs was taken as 1.0. One-way ANOVA followed by the Bonferroni *post hoc* test. (∗∗*p* < 0.01, compared with “day 2” and “day 4”). Data are shown as means ± SD (*n* = 3). See also Figures S2 and S3.

Various inflammatory cytokines are known to be involved in the onset and progression of IBD. Serum concentrations of TNF-α, IFN-γ, and IL-1β in IBD patients are higher than those of healthy subjects.^3^ Here, we investigated whether we could reproduce the inflammatory response observed in IBD patients in hiPSC-COs by exposure to the same inflammatory cytokines (Figure 2A). Colon organoids were differentiated from hiPSCs for 21 days and then treated with various cytokines. Because of the higher levels of IL-8 in the colon mucosa of IBD patients,^15^ we used *IL-8* expression, in parallel to a cytokine ELISA panel, as an indicator of the triggered inflammatory response. We searched for a combination of cytokines to increase the production of inflammatory cytokines, including IL-8. The combined treatment of hiPSC-COs with 30 ng/mL TNF-α, 30 ng/mL IFN-γ, and 10 ng/mL IL-1β for 8 days significantly increased inflammatory cytokine production (IL-6, IFN-γ-inducible protein-10 [IP-10, CXCL10], IL-8, and IL-12(p70)) (Figure 2B) and *IL-8* expression (Figure S2A; Table S2) and reduced cell viability to 52.7% (Figure S2B; Table S3). Next, we optimized the concentration of cytokines to induce inflammatory responses efficiently in hiPSC-COs. The treatment of hiPSC-COs with greater than or equal to 30 ng/mL TNF-α, 30 ng/mL IFN-γ, and 10 ng/mL IL-1β (conditions B, C, and D) for 8 days increased inflammatory cytokine concentrations (IL-6, IP-10 [CXCL10], IL-8, and IL-12(p70)) in the cell culture supernatant (Figure 2C) and *IL-8* expression (Figure S3; Table S4). However, the cell viability of hiPSC-COs was decreased to 19.6% (condition B) or 19.3% (condition C) by treatment with greater than 30 ng/mL TNF-α, 30 ng/mL IFN-γ, and 10 ng/mL IL-1β for 8 days, suggesting that high concentrations of TNF-α, IFN-γ, and IL-1β (conditions B and C) caused severe cell death in hiPSC-COs (Figure 2D; Tables S5 and S6). Cell viability of hiPSC-COs treated with lower than or equal to 30 ng/mL TNF-α, 30 ng/mL IFN-γ, and 10 ng/mL IL-1β (conditions D, E, F, G, and H) was greater than 45%. For all subsequent experiments in this study, we used a combined treatment of 30 ng/mL TNF-α, 30 ng/mL IFN-γ, and 10 ng/mL IL-1β (condition D, herein referred to as 3CK). Additionally, we investigated the optimal duration of treatment with 3CK. While 3CK treatment for 2 days did not induce cell death and inflammatory gene expression, the increase in *IL-8* expression reached a plateau at day 6 (Figure 2E; Table S7). These findings, therefore, suggested that treatment with 3CK for more than 6 days was necessary to observe a sufficient cytokine response.

### Characterization of hiPSC-COs with 3CK treatment

**Figure 3:**
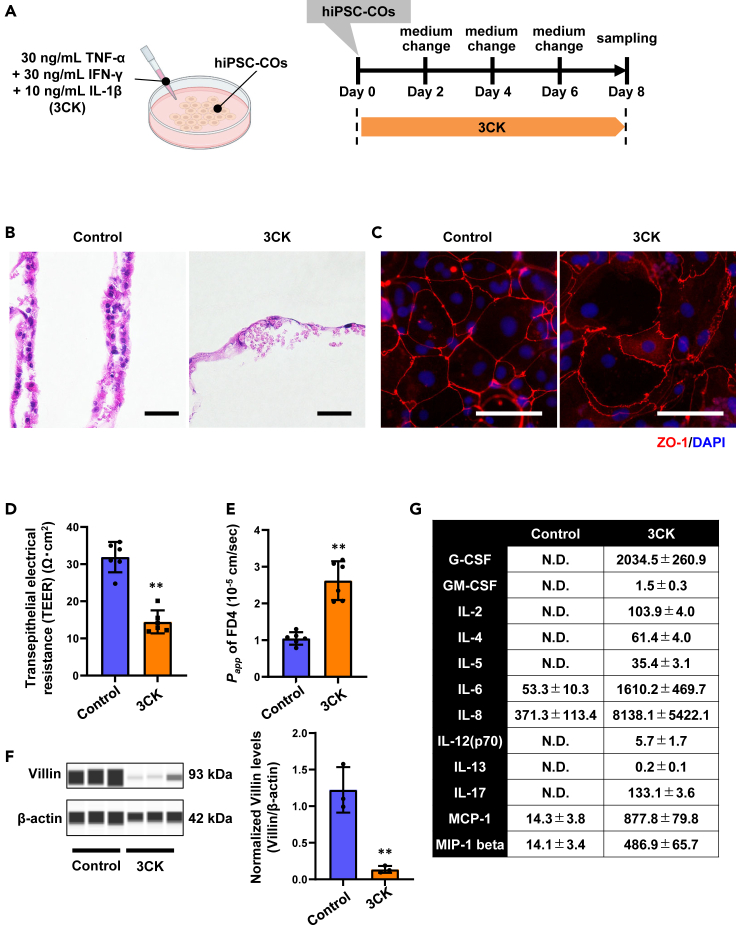
Characterization of hiPSC-COs treated with TNF-α, IFN-γ, and IL-1β (A) hiPSC-COs were treated with TNF-α (30 ng/mL), IFN-γ (30 ng/mL), and IL-1β (10 ng/mL) (referred to as 3CK) for 8 days. (B) Hematoxylin and eosin staining was performed in control and 3CK-treated hiPSC-COs. Scale bars represent 50 μm. (C) Immunofluorescence images of ZO-1 (red) in control and 3CK-treated hiPSC-COs are shown. Nuclei were counterstained with DAPI (blue). Scale bars represent 100 μm. (D) Transepithelial electrical resistance (TEER) values of hiPSC-COs treated with control or 3CK for 8 days were measured using Millicell-ERS2. Unpaired two-tailed Student’s t test (∗∗*p* < 0.01). Data are shown as means ± SD (*n* = 6). (E) The apparent permeability coefficient (*Papp*) values of FITC-Dextran (4 kDa) in hiPSC-COs treated with control or 3CK for 8 days were calculated. Unpaired two-tailed Student’s t test (∗∗*p* < 0.01). Data are shown as means ± SD (*n* = 6). (F) Protein levels of Villin in control and 3CK-treated hiPSC-COs were examined by Jess analysis. Unpaired two-tailed Student’s t test (∗∗*p* < 0.01). Data are shown as means ± SD (*n* = 3). (G) Cytokine productions in control and 3CK-treated hiPSC-COs were measured by Bio-Plex assay. Concentrations of cytokine (G-CSF, GM-CSF, IL-2, IL-4, IL-5, IL-6, IL-8, IL-12(p70), IL-13, IL-17, MCP-1, and MIP-1 beta) (pg/mL) are indicated as means ± SD (*n* = 5). N.D., not detectable. See also Figures S4 and S5.

We investigated in detail the effects of 8-day 3CK treatment on hiPSC-COs (Figure 3A). Hematoxylin and eosin (H&E) staining showed that 3CK treatment caused morphological changes, such as nuclear disappearance and cell aggregation (Figure 3B). Immunofluorescence images showed cell swelling and changes in the localization of zonula occludens protein-1 (ZO-1) (Figure 3C). To evaluate intestinal barrier function, transepithelial electrical resistance (TEER) value and apparent permeability coefficient (*Papp*) value of fluorescein isothiocyanate isomer (FITC)-Dextran (4 kDa) were examined in hiPSC-COs treated with 3CK for 8 days. TEER and *Papp* were decreased and increased in 3CK-treated hiPSC-COs, respectively, suggesting that the intestinal barrier was disrupted by 3CK treatment (Figures 3D and 3E). In addition, the protein level of Villin, an intestinal epithelial cell marker, in hiPSC-COs was decreased by 3CK treatment, suggesting that colonic epithelial cells were damaged (Figure 3F). In contrast, gene expression of *TFF3*, a goblet cell marker, in hiPSC-CO was unchanged by 3CK treatment (Figure S4A). We also found that gene expression of cytokines (*IL1B*, *IL-6*, *IL-8*, *IL18*, *IL23A*, *IL32*, and *TNF*) in hiPSC-COs was induced by 3CK treatment (Figure S4B). Consistently, cytokine production (granulocyte colony-stimulating factor [G-CSF], granulocyte-macrophage colony-stimulating factor [GM-CSF], IL-2, IL-4, IL-5, IL-6, IL-8, IL-12(p70), IL-13, IL-17, monocyte chemoattractant protein-1 [MCP-1], and macrophage inflammatory protein 1 [MIP-1] beta) was increased by 3CK treatment (Figure 3G). In particular, the production of IL-8 was increased approximately 25 times by 3CK treatment (Figure S4C). These results suggest that the inflammatory response and damage to colonic epithelial cells can be reproduced using 3CK-treated hiPSC-COs.

β-defensin, a type of antibacterial peptide, is known to play a role in biological defense. It was previously reported that the expression of human β-defensin-2 (encoded by the *defensin beta 4A* [*DEFB4A*] gene) increases during colon inflammation.^16^ We examined whether *DEFB4A* expression in hiPSC-COs changes by 3CK treatment. Gene expression of *DEFB4A* was increased approximately 121 times by 3CK treatment (Figure S4D). It has also been reported that *dual oxidase 2* (*DUOX2*) and *dual oxidase maturation factor 2* (*DUOXA2*), enzymes that generate reactive oxygen species, are highly expressed in the colon of IBD patients.^17^ Gene expression of *DUOX2* and *DUOXA2* in hiPSC-COs was increased approximately 3-fold and 5-fold, respectively, in response to 3CK treatment (Figure S4E). Furthermore, lactate dehydrogenase secretion was increased due to 3CK treatment, suggesting cytotoxicity occurred in 3CK-treated hiPSC-COs (Figure S4F). These results indicate that the biological defense response can be reproduced in 3CK-treated hiPSC-COs.

As mentioned earlier, Caco-2 cells are a cell line widely used as a colon model.^6^ Therefore, we also examine the responses of Caco-2 cells to 3CK treatment (Figure S5A). The 8-day 3CK treatment increased gene expression of inflammatory cytokines (*IL1B*, *IL-8*, and *IL23A*) (Figure S5B) and cytokine production (IL-6, IL-8, and IP-10 [CXCL10]) in Caco-2 cells (Figure S5C). When Caco-2 cells were treated with 3CK, the concentrations of IL-6 and IL-8 were 18.0 pg/mL and 923.0 pg/mL, respectively (Figure S5C). On the other hand, when hiPSC-COs were treated with 3CK, the concentrations of IL-6 and IL-8 were 1,610 pg/mL and 8,138 pg/mL, respectively (Figure 3G). These results suggest that hiPSC-COs were more sensitive to cytokines than Caco-2 cells. In addition, the gene expression of intestinal epithelial cell markers (*VIL1* and *SI*) (Figure S5D) and cell viability (Figure S5E) were unaltered. These results reveal that epithelial cell damage was not observed in Caco-2 cells treated with 3CK. Altogether, our findings suggest that hiPSC-COs represent a useful model to reproduce not only inflammatory responses but also epithelial cell damage.

### Analysis of inflammatory responses in 3CK-treated hiPSC-COs at single-cell level

**Figure 4:**
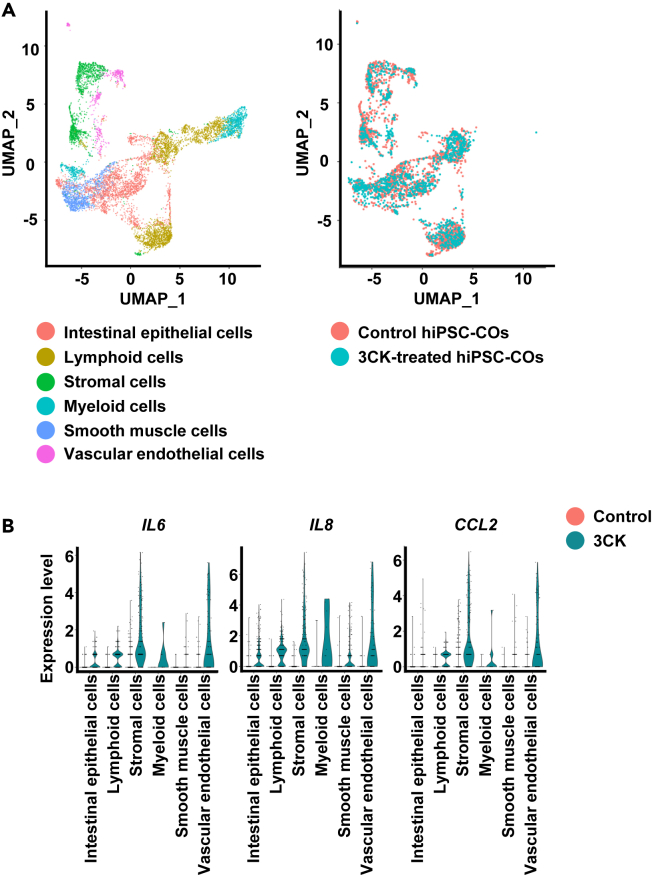
Characterization of 3CK-treated hiPSC-COs using scRNA-seq analysis hiPSC-COs were treated with TNF-α (30 ng/mL), IFN-γ (30 ng/mL), and IL-1β (10 ng/mL) (referred to as 3CK) for 8 days. (A) scRNA-seq analysis of control and 3CK-treated hiPSC-COs was performed. UMAP plots of control and 3CK-treated hiPSC-COs. (B) Violin plots show gene expression of inflammatory cytokines (*IL-6*, *IL-8*, and *CCL2*) in control and 3CK-treated hiPSC-COs. One-way ANOVA followed by Holm-Sidak *post hoc* test. *p* values for all comparisons are shown in Table S8.

We analyzed the inflammatory response in more detail by performing scRNA-seq analysis in 3CK-treated hiPSC-COs. Control and 3CK-treated hiPSC-COs were classified into 6 clusters (Figure 4A). Inflammatory cytokines (*IL-6*, *IL-8*, and *C-C motif chemokine ligand 2* [*CCL2*]) were highly expressed in stromal and vascular endothelial cell clusters in 3CK-treated hiPSC-COs (Figure 4B; Table S8), suggesting that stromal and vascular endothelial cells are the primary source of inflammatory cytokines.

### Comparison of molecular signatures in stromal cells of 3CK-treated hiPSC-COs with those of a UC patient

IBD is classified into UC and Crohn’s disease (CD). Thus, we compared gene expression profiles between the active site of colon in IBD (UC and CD) patients and 3CK-treated hiPSC-COs to determine whether our model has properties similar to UC or CD (Figure S6). From the published RNA sequencing data of IBD patient samples,^18^ we selected 10 differentially expressed genes in IBD patients (Table S9) and investigated their gene expression in 3CK-treated hiPSC-COs. Specifically, we compared the expression of these genes between IBD patients and 3CK-treated hiPSC-COs by calculating their correlation coefficients. The correlation coefficient R^2^ between CD patients and 3CK-treated hiPSC-COs was 0.2613, while the correlation coefficient R^2^ between UC patients and 3CK-treated hiPSC-COs was 0.6245. This result suggests that 3CK-treated hiPSC-COs have a gene expression profile more similar to UC patients.

We next examined UC-associated gene expression in 3CK-treated hiPSC-COs. Linggi et al. performed a meta-analysis of gene expression using colonic biopsy tissue from UC patients to select UC-specific genes.^19^ They reported that the gene expression of *DUOX2*, *chitinase 3 like 1* (*CHI3L1*), and *peptidase inhibitor 3* (*PI3*) is upregulated, and *3-hydroxy-3-methylglutaryl-CoA synthase 2* (*HMGCS2*) and UDP *glucuronosyltransferase family 2 member A3* (*UGT2A3*) expression was downregulated in the colonic biopsy tissue of UC patients. Consistently, the gene expression of *DUOX2, CHI3L1*, and *PI3* was increased in hiPSC-COs by 3CK treatment (Figure S7A), while gene expression of *HMGCS2* and *UGT2A3* was decreased in hiPSC-COs by 3CK treatment (Figure S7B). These results, taken together, suggest that 3CK-treated hiPSC-COs can replicate UC signatures.

**Figure 5:**
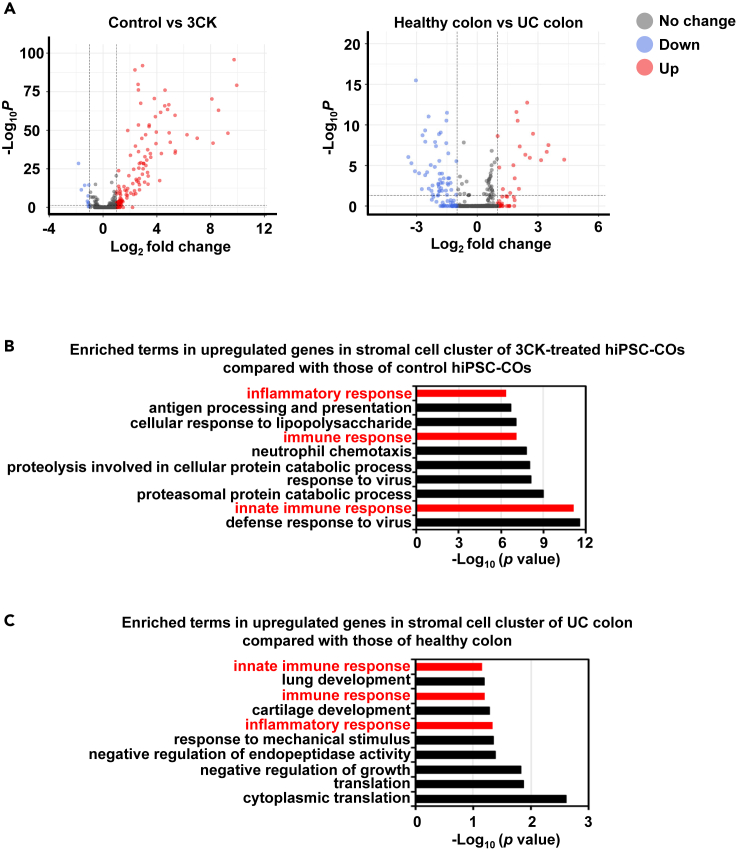
Comparative analysis between 3CK-treated hiPSC-COs and UC patient samples hiPSC-COs were treated with TNF-α (30 ng/mL), IFN-γ (30 ng/mL), and IL-1β (10 ng/mL) (referred to as 3CK) for 8 days. (A) Gene expression profiles in the stromal cell cluster of 3CK-treated hiPSC-COs were compared with active site of colon tissue from a UC patient.^20^ The left panel shows a volcano plot of differentially expressed genes identified between control and 3CK-treated hiPSC-COs (log_2_ fold change >1, adjusted *p* value [padj] < 0.05). The right panel shows a volcano plot of differentially expressed genes identified between the healthy colon and UC colon (log_2_ fold change >1, adjusted *p* value [padj] < 0.05). Red and blue dots represent up- and downregulated genes, respectively, in 3CK-treated hiPSC-COs (left) and UC colon (right). (B) GO enrichment analysis of upregulated genes in the stromal cell cluster of 3CK-treated hiPSC-COs compared with those of control hiPSC-COs. (C) GO enrichment analysis of upregulated genes in the stromal cell cluster of UC colon compared with those of healthy colon. Common terms between stromal cells of 3CK-treated hiPSC-COs (B) and UC colon (C) are shown in red. See also Figures S6 and S7.

We also compared the scRNA-seq data of 3CK-treated hiPSC-COs and the colon of a UC patient (Figures S7C and S7D). As shown in Figure 5A, the expression of 110 genes and 17 genes was upregulated in the stromal cell cluster of 3CK-treated hiPSC-COs and UC colon, respectively. Gene ontology (GO) enrichment analysis showed that genes for “inflammatory response,” “immune response,” and “innate immune response” were enriched in upregulated genes in 3CK-treated hiPSC-COs compared with control hiPSC-COs (Figure 5B). Similarly, genes related to these three terms were also enriched in upregulated genes in the UC colon compared with the healthy colon (Figure 5C). These results indicate that the inflammatory responses in stromal cells of the UC colon can be recapitulated using 3CK-treated hiPSC-COs.

### Evaluation of the UC therapeutic agents using hiPSC-COs

**Figure 6:**
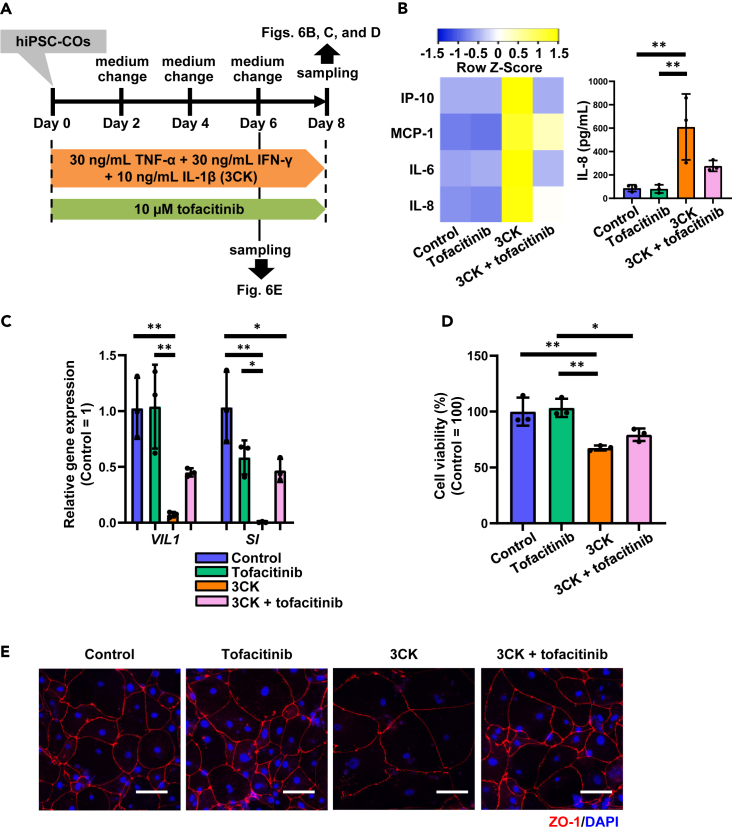
Evaluation of tofacitinib using 3CK-treated hiPSC-COs (A) In the presence of TNF-α (30 ng/mL), IFN-γ (30 ng/mL), and IL-1β (10 ng/mL) (referred to as 3CK), hiPSC-COs were treated with or without tofacitinib (10 μM) for 6 or 8 days. (B) Concentrations of cytokines secreted from control, tofacitinib-, 3CK-, or 3CK and tofacitinib-treated hiPSC-COs were examined by a bead-based multiplex immunoassay (LEGENDplex analysis). Heatmap shows the average concentration of cytokines (IP-10 [CXCL10], MCP-1, IL-6, and IL-8) in the culture supernatants of control, 3CK-, or 3CK + tofacitinib-treated hiPSC-COs (left). Amounts of IL-8 secreted by control, tofacitinib-, 3CK-, or 3CK + tofacitinib-treated hiPSC-COs are shown in the right. One-way ANOVA followed by the Tukey *post hoc* test (∗∗*p* < 0.01). Data are shown as means ± SD (*n* = 3). (C) Gene expression of *VIL1* and *SI* in control, tofacitinib-, 3CK-, or 3CK + tofacitinib-treated hiPSC-COs was examined by RT-qPCR. Gene expression in control hiPSC-COs was taken as 1.0. One-way ANOVA followed by the Tukey *post hoc* test (∗*p* < 0.05, ∗∗*p* < 0.01). Data are shown as means ± SD (*n* = 3). (D) The cell viability of control, tofacitinib-, 3CK-, or 3CK + tofacitinib-treated hiPSC-COs was examined by WST-8 assay. One-way ANOVA followed by the Tukey *post hoc* test (∗*p* < 0.05, ∗∗*p* < 0.01). Data are shown as means ± SD (*n* = 3). (E) Immunofluorescence images of ZO-1 (red) in control, tofacitinib-, 3CK-, or 3CK- and tofacitinib-treated hiPSC-COs are shown. Nuclei were counterstained with DAPI (blue). Scale bars represent 100 μm. See also Figure S8.

To validate whether our UC model could be used to evaluate IBD therapeutics, we examined the efficacy of tofacitinib, a JAK inhibitor used to treat UC.^21^ We treated hiPSC-COs with 3CK and 10 μM tofacitinib simultaneously (Figure 6A). We confirmed that 10 μM tofacitinib showed no cytotoxicity in hiPSC-COs (Figure S8A) and, more importantly, that it reversed the 3CK treatment-mediated increase of gene expression and secretion of inflammatory cytokine in hiPSC-COs (Figures 6B and S8B). 3CK treatment-mediated reductions in gene expression of colonic epithelial markers, *VIL1* and *SI*, in hiPSC-COs were recovered by tofacitinib treatment (Figure 6C). Cell viability of 3CK-treated and 3CK- and tofacitinib-treated hiPSC-COs was 67.5% and 79.3%, respectively. (Figure 6D). Furthermore, immunofluorescence images showed that 3CK-induced cell swelling and changes in the ZO-1 localization were rescued by tofacitinib treatment (Figure 6E). We investigated whether the therapeutic effects would appear even if we started treating hiPSC-COs with tofacitinib 24 h after initiating 3CK-mediated inflammatory responses (Figure S8C). The elevated gene expression of inflammatory cytokines was suppressed by tofacitinib treatment (Figure S8D). These results, together, suggest that the therapeutic effects of tofacitinib can be evaluated using hiPSC-COs and highlight the potential of our UC model to serve as a UC drug development platform.

## Discussion

Here, we generated colon organoids containing colonic epithelial cells and non-epithelial cells differentiated from hiPSCs. HiPSCs were differentiated into definitive endoderm and mesoderm cells simultaneously, which were further differentiated into colonic epithelial cells and non-epithelial cells, including smooth muscle, stromal, and vascular endothelial cells. By examining combinations of cytokines, we succeeded in reproducing UC characteristics in hiPSC-COs. In 3CK-treated hiPSC-COs, we revealed that not only are inflammatory cytokines released from stromal and vascular endothelial cells but damage to colonic epithelial cells also occurs. In addition, inflammatory response-related genes were enriched in stromal cells of both 3CK-treated hiPSC-COs and UC patient’s colon. Furthermore, we confirmed that this UC model can be used to evaluate the efficacy of potential therapeutics.

Intestinal organoids are utilized to study the molecular pathophysiology of IBD, evaluate the therapeutics for IBD, and explore the potential of cell therapy for IBD.^22^^,^^23^^,^^24^^,^^25^^,^^26^^,^^27^ However, most of the previous intestinal organoids were composed of only colonic epithelial cells, making it difficult to accurately model the interactions between colonic epithelial cells and non-epithelial cells. On the other hand, our hiPSC-COs consist of colonic epithelial and non-epithelial cells. In hiPSC-COs treated with 3CK, inflammatory responses, such as increased *IL-8* expression, intestinal epithelial cell damage such as epithelial barrier disruption, and decreased expression of intestinal epithelial cell markers were observed (Figure 3). On the other hand, the inflammatory responses in 3CK-treated Caco-2 cells, a homogeneous population of colonic epithelial cells, were weaker than those in hiPSC-COs cells, and 3CK treatment-mediated intestinal epithelial cell damage was not observed in Caco-2 cells (Figure S5). Consistent with the data obtained in the colon of UC patients, analysis of scRNA-seq in 3CK-treated hiPSC-COs revealed that inflammatory response-related genes are enriched in stromal cells in response to 3CK treatment. Therefore, compared with a conventional colon model composed of colonic epithelial cells alone, hiPSC-COs, with both colonic epithelial and non-epithelial cells, are more suitable for recapitulating the pathophysiology of UC.

It is known that focal cell loss, erosions, and ulcers are observed in the colon of UC patients, followed by repeated recovery and recurrence of inflammation.^28^ Although our 3CK-treated hiPSC-COs exhibited focal cell loss, they did not completely recapitulate the cell-type composition of the colon of UC patients and display histological features like erosions and ulcers. In addition, recovery and recurrence of UC were not recapitulated in hiPSC-COs. Further improvement of hiPSC-COs is necessary to faithfully recapitulate the pathophysiology of UC. For example, colonic epithelial cells, lamina propria, and submucosa are damaged in ulcers.^29^ Therefore, it will be difficult to mimic ulcers unless a colon model that includes lamina propria and submucosa is established. Since intestinal tissue with lamina propria and submucosa can be generated by transplanting intestinal organoids under the kidney capsule of immunodeficient mice,^30^ it may be possible to reproduce ulcers of UC by using such intestinal chimeric mice.

In addition to excessive inflammatory cytokines, genetic predisposition^31^ and gut microbiome^32^ are also involved in UC. McGovern et al. performed a meta-analysis using two distinct genome-wide association study datasets from UC patients and identified 27 UC susceptibility loci, including immunoglobulin receptor genes.^31^ Although we used iPSCs established from healthy individuals, by using iPSCs from patients with mutations in the UC-associated genes, it may be possible to examine with finer molecular details the UC pathophysiology caused by such mutations. By a multi-omics analysis of stool, colon biopsy, and blood obtained from IBD patients, Lloyd-Price et al. found that the relative abundance of gut bacteria and gut bacteria-derived metabolites differed between individuals with CD, UC, and non-IBD participants.^32^ In the future, by co-culturing hiPSC-COs with intestinal bacteria isolated from IBD patients, it will be possible to analyze in more detail the relationship between IBD pathophysiology and dysbiosis. We expect that elucidating the mechanisms underlying the initiation and exacerbation of IBD due to excessive inflammatory cytokines, genetic mutations, and gut microbiome will pave the way for the development of effective therapeutic drugs.

### Limitations of the study

In 3CK-treated hiPSC-COs, the concentrations of multiple cytokines, including IL-8, IL-6, and IP-10 (CXCL10), were increased. Induced production of these cytokines suggests that 3CK-treated hiPSC-COs were able to recapitulate the colon of UC patients partially. However, the concentrations of 3CK in this study (30 ng/mL TNF-α, 30 ng/mL IFN-γ, and 10 ng/mL IL-1β) are much higher than those in the blood of IBD patients (3,000 pg/mL TNF-α, 900 pg/mL IFN-γ, and 200 pg/mL IL-1β).^3^ Low concentrations of 3CK did not sufficiently induce a sufficient inflammatory response and cellular damage in hiPSC-COs, suggesting that 3CK treatment alone cannot recapitulate the complete pathophysiology of UC patients. It has been reported that low concentrations of 3CK induced the expression of multiple proteins that promote inflammation in the presence of bacterial lipopolysaccharide.^33^ Therefore, in the future, we will need to use factors other than 3CK to reproduce the pathophysiology of UC patients. Additionally, IL-13 secreted from lamina propria immune cells of UC patients is known to impair the colonic epithelial barrier function.^34^ In the future, we believe that it would be possible to more accurately recapitulate the pathophysiology of UC by treating hiPSC-COs with cytokines and bacterial component or co-culturing hiPSC-COs with immune cells that produce a variety of IBD-related inflammatory cytokines, including IL-13.

## Resource availability

### Lead contact

Further information and requests for resources and reagents should be directed to and will be fulfilled by the lead contact, Dr. Kazuo Takayama (kazuo.takayama@cira.kyoto-u.ac.jp).

### Materials availability

All materials are available from the corresponding author on reasonable request.

### Data and code availability

• Single-cell RNA sequencing data have been deposited at GEO (accession number: GSE244981) and are publicly available as of the date of publication. Other data and images that support the findings of this study are available on request from the lead contact.
• This paper does not report original code.
• Any additional information required to reanalyze the data reported in this paper is available from the lead contact upon request.

## Acknowledgments

We thank Dr. Kelvin Hui (Kyoto University) for critical reading of the manuscript; Ms. Junko Kuwahara, Dr. Hiromi Dohi, and Dr. Masaki Nomura (CiRA Foundation) for technical assistance with the scRNA-seq experiments; and Ms. Natsumi Mimura, Ms. Ayaka Sakamoto, Ms. Naoko Yasuhara, and Ms. Shiho Morimoto (Kyoto University) for technical assistance. Figure 3A and graphical abstract were created using BioRender (https://biorender.com). Figures 2B, 2C, and 6B were created using Heatmapper (http://heatmapper.ca/expression/). This research was supported by the iPS Cell Research Fund, the 10.13039/100009619Japan Agency for Medical Research and Development (AMED) (JP21gm1610005, JP23bm1323001), the 10.13039/501100002241Japan Science and Technology Agency (JST) (ACT-X, JPMJAX222A), and JSPS Core-to-Core Program (A. Advanced Research Networks) JPJSCCA20240006.

## Author contributions

F.Y.: research design, cell culture, analysis, and manuscript preparation. S.D.: research design, manuscript preparation, and funding acquisition. Y.W.: scRNA-seq analysis and manuscript preparation. K.T.: research design, manuscript review and editing, funding acquisition, and final approval. F.Y. and S.D. contributed equally.

## Declaration of interests

The authors declare no competing interests.

## STAR★Methods

### Key resources table

**Table:** 

REAGENT or RESOURCE	SOURCE	IDENTIFIER
**Antibodies**		

Cytokeratin 20 Polyclonal antibody	Proteintech	Cat# 17329-1-AP; RRID: AB_2133592
Rabbit polyclonal anti-ZO-1	Cell Signaling	Cat# 5406; RRID: AB_1904187
Mouse monoclonal anti-β-Actin	Sigma-Aldrich	Cat# A5441; RRID: AB_476744
Rabbit monoclonal anti-Villin	Abcam	Cat# ab109516; RRID: AB_10861582
Cytokeratin 20 Monoclonal antibody	Proteintech	Cat# 60183-1-Ig; RRID: AB_10858399
Alexa 488-conjugated anti- rabbit IgG antibody	Thermo Fisher Scientific	Cat# A-21206; RRID: AB_2535792
Alexa 594-conjugated anti-rabbit IgG antibody	Thermo Fisher Scientific	Cat# A11012; RRID: AB_2534079

**Chemicals, peptides, and recombinant proteins**		

i-Matrix-511 silk	Nippi	Cat# 892021
StemFit AK02N medium	Ajinomoto Healthy Supply	Cat# RCAK02N
TrypLE Select Enzyme	Thermo Fisher Scientific	Cat# 12563029
Y-27632	FUJIFILM Wako Pure Chemical	Cat# 034-24024
Eagle’s Minimum Essential Medium	FUJIFILM Wako Pure Chemical	Cat# 051-07615
1× GlutaMAX	Thermo Fisher Scientific	Cat# 35050-079
TNF-α	PeproTech	Cat# 300-01A
IFN-γ	PeproTech	Cat# 300-02
IL-1β	PeproTech	Cat# 200-01B
tofacitinib	Selleck Chemicals	Cat# S2789
Matrigel Growth Factor Reduced Basement Membrane	Corning	Cat# 354230
Activin A	R&D Systems	Cat# 338-AC
1× B-27 Supplement Minus Vitamin A	Thermo Fisher Scientific	Cat# 12587001
Advanced DMEM/F12	Thermo Fisher Scientific	Cat# 12634010
Dulbecco’s Modified Eagle Medium	FUJIFILM Wako Pure Chemical	Cat# 044-29765
1×MEM Non-Essential Amino Acids Solution	Thermo Fisher Scientific	Cat# 11140050
1× N2 Supplement with Transferrin	FUJIFILM Wako Pure Chemical	Cat# 141-08941
Fibroblast growth factor 2 (FGF2)	Katayama Chemical Industries	Cat# 160-0010-3
Epidermal growth factor (EGF)	PeproTech	Cat# AF-100-15
Forskolin	FUJIFILM Wako Pure Chemical	Cat# 063-02193
PD98059	Selleck Chemicals	Cat# S1177
5-aza-2′-deoxycytidine	FUJIFILM Wako Pure Chemical	Cat# 018-20941
A-83-01	FUJIFILM Wako Pure Chemical	Cat# 035-24113
Noggin	PeproTech	Cat# 120-10C
R-spondin 1	PeproTech	Cat# 120-38
Gastrin	Tocris Bioscience	Cat# 3006/1
IGF1	PeproTech	Cat# 100-11
FGF7	PeproTech	Cat# AF-100-19
ISOGENE	NIPPON GENE	Cat# 319-90211
SYBR Green PCR Master Mix	Thermo Fisher Scientific	Cat# 4385614
4% paraformaldehyde	FUJIFILM Wako Pure Chemical	Cat# 163-20145
DAPI Solution	Nacalai Tesque	Cat# 19178-91
HistoVT One	Nacalai Tesque	Cat# 06380-05
Tris Buffered Saline with 0.1 %-Detergent	Nacalai Tesque	Cat# 12750-81
Blocking One Histo	Nacalai Tesque	Cat# 06349-64
ProLong™ Glass Antifade Mountant with NucBlue™ Stain	Nacalai Tesque	Cat# P36985
RIPA buffer	Thermo Fisher Scientific	Cat# 89900
Halt™ Protease Inhibitor Cocktail	Thermo Fisher Scientific	Cat# 78438
12–230 kDa Separation Module	ProteinSimple	Cat# SM-W001
FITC-dextran (4 kDa)	Sigma-Aldrich	Cat# FD4
HBSS	Thermo Fisher Scientific	Cat# 14025076

**Critical commercial assays**		

Cell Counting Kit-8	Dojindo	Cat# 343-07623
Quantikine® ELISA Human IL 8/CXCL8 Immunoassay	R&D Systems	Cat# D8000C
LEGENDplex Human Anti-Virus Response Panel	BioLegend	Cat# 740 390
Bio-Plex Pro Human Cytokine 17-Plex Panel	Bio-Rad	Cat# M5000031YV
Superscript VILO cDNA Synthesis Kit	Thermo Fisher Scientific	Cat# 11754250
single-cell 3′ reagent kits v3.1	10x Genomics	Cat# 1000269
LDH-Glo™ Cytotoxicity Assay	Promega	Cat# J2381
FITC Annexin V Apoptosis Detection Kit with PI	BioLegend	Cat# 640914

**Deposited data**		

scRNA-seq data	This paper	GEO: GSE244981
scRNA-seq datasets of human healthy colons	Garrido-Trigo et al.^28^	GSM6614353
scRNA-seq datasets of human UC colons	Garrido-Trigo et al.^28^	GSM6614356

**Experimental models: Cell lines**		

Human: 1383D6 iPS cells	Nakagawa et al.^22^	N/A
Human: Caco-2 cells	American Type Culture Collection	Cat# HTB-37

**Oligonucleotides**		

Primers for RT-qPCR analysis, see Table S1	This paper	N/A

**Software and algorithms**		

Compass for Simple Western software	ProteinSimple	https://www.bio-techne.com/resources/instrument-software-download-center/compass-software-simple-western
Cell Ranger pipeline (version 7.1.0)	10x Genomics	https://www.10xgenomics.com/jp/support/software/cell-ranger/latest
Seurat (version 4.3.3)	Butler et al.^30^	https://satijalab.org/seurat/
scType	Ianevski et al.^32^	https://sctype.app/

**Other**		

QuantStudio 1 real-time PCR system	Thermo Fisher Scientific	N/A
QuantStudio 3 real-time PCR system	Thermo Fisher Scientific	N/A
BZ-X710	Keyence Corporation	N/A
Millicell-ERS2	Merck Millipore	N/A
Jess	ProteinSimple	N/A
Envision 2104 Multilabel Reader	PerkinElmer	N/A
MACSQuant Analyzer 10	Miltenyi Biotech	N/A
NovaSeq 6000	Illumina	N/A

### Experimental model and study participant details

#### Human induced pluripotent stem cells

The hiPSC line, 1383D6, which was previously established from peripheral blood mononuclear cells of a healthy male (provided by Dr. Masato Nakagawa, Kyoto University),^35^ was maintained on 0.5 μg/cm^2^ recombinant human laminin 511 E8 fragments (i-Matrix-511 silk, Cat# 892021, Nippi) with StemFit AK02N medium (Cat# RCAK02N, Ajinomoto) containing 10 μM Y-27632 (Cat# 034-24024, FUJIFILM Wako Pure Chemical). For cell passaging, hiPSC colonies were treated with TrypLE Select Enzyme (Cat# 12563029, Thermo Fisher Scientific) for 10 min at 37°C. STR analysis was performed to authenticate hiPSC line. No mycoplasma contamination was confirmed using the MycoAlert Mycoplasma Detection Kit (Cat# LT07-318, Lonza).

#### Caco-2 cells

The human colorectal adenocarcinoma cell line, Caco-2 cell (Cat# HTB-37), was obtained from the American Type Culture Collection (ATCC). Caco-2 cells were maintained with Eagle’s Minimum Essential Medium (Cat# 051-07615, FUJIFILM Wako Pure Chemical) supplemented with 10 % fetal bovine serum (Cat# 35-010-CV, Corning), 1×GlutaMAX (Cat# 35050-079, Thermo Fisher Scientific), and 1×penicillin-streptomycin (Cat# 26239-42, Cat# 32204-92, Nacalai Tesque). For cell passaging, Caco-2 cells were treated with TrypLE Select Enzyme for 10 min at 37°C. STR analysis was performed to authenticate this cell line. No mycoplasma contamination was confirmed using the MycoAlert Mycoplasma Detection Kit (Cat# LT07-318, Lonza).

### Method details

#### Three-cytokines (3CK) treatment experiments

HiPSC-COs and Caco-2 cells were treated with TNF-α (Cat# 300-01A, PeproTech), IFN-γ (Cat# 300-02, PeproTech), and IL-1β (Cat# 200-01B, PeproTech) for 6 or 8 days.

#### Tofacitinib treatment experiments

HiPSC-COs were treated with TNF-α, IFN-γ, and IL-1β for 6 or 8 days in the presence or absence of tofacitinib (Cat# S2789, Selleck Chemicals).

#### hiPSC-CO generation

To generate hiPSC-COs, hiPSCs were seeded on Matrigel® Growth Factor Reduced Basement Membrane (Cat# 354230, Corning)-coated cell culture plates (2.0×10^5^ cells/4 cm^2^) and cultured for 2 days. From day 0 to 3, hiPSCs were treated with 100 ng/mL activin A (Cat# 338-AC-01M, R&D Systems) and 10 μM Y-27632 in 1:1 mixture of advanced DMEM/F12 (Cat# 12634-010, Thermo Fisher Scientific) and Dulbecco’s Modified Eagle Medium (Cat# 044-29765, FUJIFILM Wako Pure Chemical) supplemented with 1 % fetal bovine serum, 1×B-27 Supplement Minus Vitamin A (Cat# 12587001, Thermo Fisher Scientific), 1×MEM Non-Essential Amino Acids Solution (Cat# 11140050, Thermo Fisher Scientific), 1×N2 Supplement with Transferrin (Cat# 141-08941, FUJIFILM Wako Pure Chemical), 1×GlutaMAX, and 1×penicillin-streptomycin. From day 3 to 7, cells were treated with 100 ng/mL FGF2 (Cat# 160-0010-3, Katayama Chemical Industries) in advanced DMEM/F12 supplemented with 1 % fetal bovine serum, 1×GlutaMAX, and 1×penicillin-streptomycin.

To generate organoids, cells were embedded in Matrigel Growth Factor Reduced Basement Membrane. For colonic differentiation, cells were treated with 20 ng/mL EGF (Cat# AF-100-15, PeproTech), 30 μM Forskolin (Cat# 063-02193, FUJIFILM Wako Pure Chemical), 20 μM PD98059 (Cat# S1177, Selleck Chemicals), 5 μM 5-Aza-2′-Deoxycytidine (Cat# 018-20941, FUJIFILM Wako Pure Chemical), and 0.5 μM A-83-01 (Cat# 035-24113, FUJIFILM Wako Pure Chemical) in advanced DMEM/F12 supplemented with fetal bovine serum, 1×GlutaMAX, 1×MEM Non-Essential Amino Acids Solution, 1×B-27 Supplement Minus Vitamin A, 1×N2 Supplement with Transferrin (Holo), and 1×penicillin-streptomycin for 6 days. On day 13 of the differentiation, hiPSC-COs were recovered from Matrigel gel. These organoids were partially dissociated, and then hemisphere organoids were plated to thin Matrigel-coated multi-well plates. To promote colonic maturation, cells were treated with Wnt3a-conditioned medium, 50 ng/mL Noggin (Cat# 120-10C, PeproTech), 100 ng/mL R-spondin 1 (Cat# 120-38, PeproTech), 10 ng/mL Gastrin (Cat# 3006/1, Tocris Bioscience), 20 ng/mL EGF, 20 ng/mL FGF2, 10 ng/mL IGF1 (Cat# 100-11, PeproTech), 1 μM A83-01, and 10 μM Y27632 in advanced DMEM/F12 supplemented with 1 % fetal bovine serum, 1×GlutaMAX, and 1×penicillin-streptomycin for 8 days. Finally, cells were treated with 20 ng/mL EGF, 20 ng/mL FGF2, 10 ng/mL FGF7 (Cat# AF-100-19, PeproTech), 1 μM A83-01, and 10 μM Y27632 in advanced DMEM/F12 supplemented with 1 % fetal bovine serum, 1×GlutaMAX, and 1×penicillin-streptomycin for 4 days.

#### RT-qPCR

Total RNA was isolated using ISOGENE (Cat# 319-90211, NIPPON GENE). cDNA was synthesized using 500 ng of total RNA with the Superscript VILO cDNA Synthesis Kit (Cat# 11754250, Thermo Fisher Scientific). RT-qPCR was performed with SYBR Green PCR Master Mix (Cat# 4385614, Thermo Fisher Scientific) using a QuantStudio 1 or QuantStudio 3 Real-Time PCR System (Thermo Fisher Scientific). Relative quantification of target mRNA levels was performed using the 2^-ΔΔCT^ method. Values were normalized to the housekeeping gene *glyceraldehyde 3-phosphate dehydrogenase* (*GAPDH*). Primer sequences are summarized in Table S10.

#### Immunofluorescence staining of hiPSC-COs cultured in multi-well plates

For immunofluorescence staining of undifferentiated hiPSCs (CK20) and hiPSC-COs (ZO-1) cultured in multi-well plates, organoids were fixed with 4% paraformaldehyde (Cat# 163-20145, FUJIFILM Wako Pure Chemical). Cells were blocked with PBS containing 10% fetal bovine serum, 2% bovine serum albumin, and 0.2% Triton X-100 at room temperature for 45 min. Then, cells were incubated with a primary antibody for 2 h at room temperature and then with a secondary antibody at room temperature for 1 h. Nuclei were counterstained with DAPI Solution (Cat# 19178-91, Nacalai Tesque) and analyzed using the BZ-X710 system (Keyence Corporation). Antibodies used for immunofluorescence staining are summarized in Table S11.

#### Immunofluorescence staining on frozen sections of hiPSC-COs

For immunofluorescence staining on frozen sections of hiPSC-COs (CK20), organoids were fixed with 4% paraformaldehyde. Fixed hiPSC-COs were used to prepare the frozen section. For antigen retrieval, sections were processed by heating at 70°C in HistoVT One (Cat# 06380-05; Nacalai Tesque) for 30 min. Sections were permeabilized using Tris Buffered Saline with 0.1 %-Detergent (Cat# 12750-81, Nacalai Tesque) and blocked using Blocking One Histo (Cat# 06349-64, Nacalai Tesque) for 10 min. Sections were incubated in Tris Buffered Saline with 0.1 %-Detergent with a primary antibody for 2 h at room temperature and then with a secondary antibody for 45 min. Sections were washed with PBS and incubated in Tris Buffered Saline with 0.1 %- Detergent containing DAPI Solution for 5 min. Sections were finally mounted with ProLong™ Glass Antifade Mountant with NucBlue™ Stain (Cat# P36985, Nacalai Tesque) and analyzed using the BZ-X710 system (Keyence Corporation). Antibodies used for immunofluorescence staining are summarized in Table S11.

#### H&E staining

HiPSC-COs harvested and used to prepare paraffin sections after fixation with 4% paraformaldehyde for 15 min. Paraffin-embedded tissue section and H&E staining were carried out by the Applied Medical Research Laboratory.

#### ELISA

IL-8 concentrations in the culture supernatants of hiPSC-COs were measured using Quantikine® ELISA Human IL 8/CXCL8 Immunoassay (Cat# D8000C, R&D Systems). The ELISA kit was used according to the manufacturer’s protocol.

#### Enzyme-linked immunosorbent assay

Cytokine and chemokine concentrations in the culture supernatants of hiPSC-COs were measured using a LEGENDplex Human Anti-Virus Response Panel (Cat# 740 390, BioLegend). Flow cytometry was performed according to the manufacturer’s instructions using MACSQuant Analyzer 10 (Miltenyi Biotec). In Figure 3G, the concentrations of cytokines and chemokines in the culture supernatants of hiPSC-COs were also measured using Bio-Plex Pro Human Cytokine 17-Plex Panel (Cat# M5000031YV, Bio-Rad) according to the manufacturer’s protocol.

#### Jess analysis

HiPSC-COs were lysed in RIPA buffer (Cat# 89900, Thermo Fisher Scientific) containing a Halt™ Protease Inhibitor Cocktail (100X) (Cat# 78438, Thermo Fisher Scientific). After the centrifugation, the supernatants were collected. Antibody-based protein quantification was performed using a Jess system (ProteinSimple) with 12-230 kDa Separation Module (Cat#SM-W001, ProteinSimple) as instructed. Antibodies used for this analysis are summarized in Table S12. Data were analyzed and visualized using Compass for Simple Western software (ProteinSimple).

#### Lactate dehydrogenase (LDH) release

To evaluate LDH release, the culture supernatants of colon organoids were collected. Collected supernatants were analyzed using LDH-Glo™ Cytotoxicity Assay (Cat# J2381, Promega) according to the manufacturer’s instructions.

#### WST-8 assay

Cell viability was assessed by Cell Counting Kit-8 (Cat# 343-07623, Dojindo) according to the manufacturer’s protocol. Cell viability without cytokines or drugs was taken as 100%.

#### Transepithelial electrical resistance (TEER) measurement

HiPSC-COs were seeded on 6.5 mm Transwell with 0.4 μm Pore Polycarbonate Membrane Insert (Cat# 3413, Corning) at 1.5 x 10^5^ cells/cm^2^. Cells were cultured in the presence or absence of 3CK for 8 days. TEER values were measured by Millicell-ERS2 (Merck Millipore). The TEER value was calculated by the following formula: (resistance of experimental wells − resistance of blank wells) × 0.33 (the area of the cell culture insert).

#### Intestinal permeability assays

To evaluate barrier function, *Papp* value of FITC-dextran (4 kDa; Cat# FD4, Sigma-Aldrich) was calculated. hiPSC-COs, which were seeded on 6.5 mm Transwell with 0.4 μm Pore Polycarbonate Membrane Insert at 1.5 x 10^5^ cells/cm^2^, were preincubated with HBSS (Cat# 14025076, Thermo Fisher Scientific) for 15 min. Afterward, 100 μL HBSS with or without 1 mg/mL FITC-dextran was added into the apical or basolateral chambers, respectively. After 30 min, culture supernatant was collected, and the fluorescence (Ex./Em. 490/520 nm) was measured using Envision 2104 Multilabel Reader (PerkinElmer). The *Papp* value was calculated according to the following equation:

$$P_{app}\;\left(\text{cm}/s\right)=\frac{\text{dQ}/\text{dt}}{A\times C_{0}}$$

In which dQ/dt is the amount of the FITC-dextran transported to the basolateral chamber over time, A is the surface area of the membrane, and C_0_ is the initial concentration of FITC-dextran in the apical chamber.

#### Apoptosis assay

The percentage of apoptotic and necrotic cells were measured using FITC Annexin V Apoptosis Detection Kit with PI (Cat# 640914, BioLegend). Briefly, hiPSC-COs treated with or without 3CK for 2 days were washed with PBS and incubated in TrypLE Select Enzyme for 10 min at 37°C. Collected cells were stained with FITC Annexin V and Propidium Iodide Solution according to the manufacturer’s instructions, followed by analysis using MACSQuant Analyzer 10.

#### Single-cell RNA sequencing

hiPSC-COs treated with or without 3CK were dissociated and the cell concentration was adjusted to 1,000 cells/μL. Each single-cell suspension containing approximately 8.3 × 10^3^ cells, was loaded onto a 10xGenomics chromium chip with enzyme mix, gel beads, and oils to generate GMES. Subsequently, reverse transcription and cDNA amplification were conducted according to the manufacturer’s instructions for single-cell 3′ reagent kits v3.1 (10x Genomics). cDNA libraries were sequenced on the NovaSeq 6000 platform (Illumina). The 10X Genomics Cell Ranger pipeline (version 7.1.0) was used to perform sample demultiplexing, alignment to the GRCh38-2020-A human reference genome, barcode/UMI processing, and gene counting for each cell. Gene count matrix data were processed as follows below. Briefly, the low-quality cells were excluded based on the following criteria: (I) number of detected genes ≥200, (II) number of UMI molecules detected within a cell ≤100,000, and (III) percentage of mitochondrial genes ≤65 %. Filtered unique molecular identifiers (UMI) feature-barcode matrices were processed with Seurat (version 4.3.3).^36^ Gene count matrices were normalized and scaled by 10,000. Dimensionality reduction with principal component analysis was performed using a set of 2,000 top variable genes (Seurat::FindVariableFeatures).^37^ Clustering was performed with 20 principal components, a k-nearest neighbor value of 20, and the Louvain clustering algorithm. Cell type annotation was performed by scType.^38^ The accession number for single-cell RNA sequencing data reported in this study is GSE244981.

scRNA-seq datasets of healthy and UC colons were obtained from a public database (GSM6614353 and GSM6614356, respectively).^34^ Healthy colon data were collected from the sigmoid colon of a 66-year-old woman. UC patient colon data were collected from the active site of the rectum of a 33-year-old woman.

### Quantification and statistical analysis

Statistical significance was evaluated by unpaired two-tailed Student’s *t*-test or one-way analysis of variance (ANOVA) followed by Holm-Sidak post-hoc tests, Dunnett’s post hoc tests, Bonferroni post-hoc tests, or Tukey’s post hoc tests (∗*p*<0.05, ∗∗*p*<0.01). Statistical analyses were performed using GraphPad Prism 9. Details are described in the figure legends. Data are representative of three independent experiments.
