# Supplementary material for: Establishment of an ulcerative colitis model using colon organoids derived from human induced pluripotent stem cells
Source: iScience. 2024 Sep 26;27(10):111049. doi: 10.1016/j.isci.2024.111049 (PMC11492162; doi:10.1016/j.isci.2024.111049)
Supplement: Document S1. Figures S1–S8 and Tables S1–S12 [file mmc1.pdf]

iScience, Volume *27*

Supplemental information

Establishment of an ulcerative colitis model using colon organoids derived from human induced pluripotent stem cells

Fuki Yokoi, Sayaka Deguchi, Yukio Watanabe, and Kazuo Takayama

Figure S1

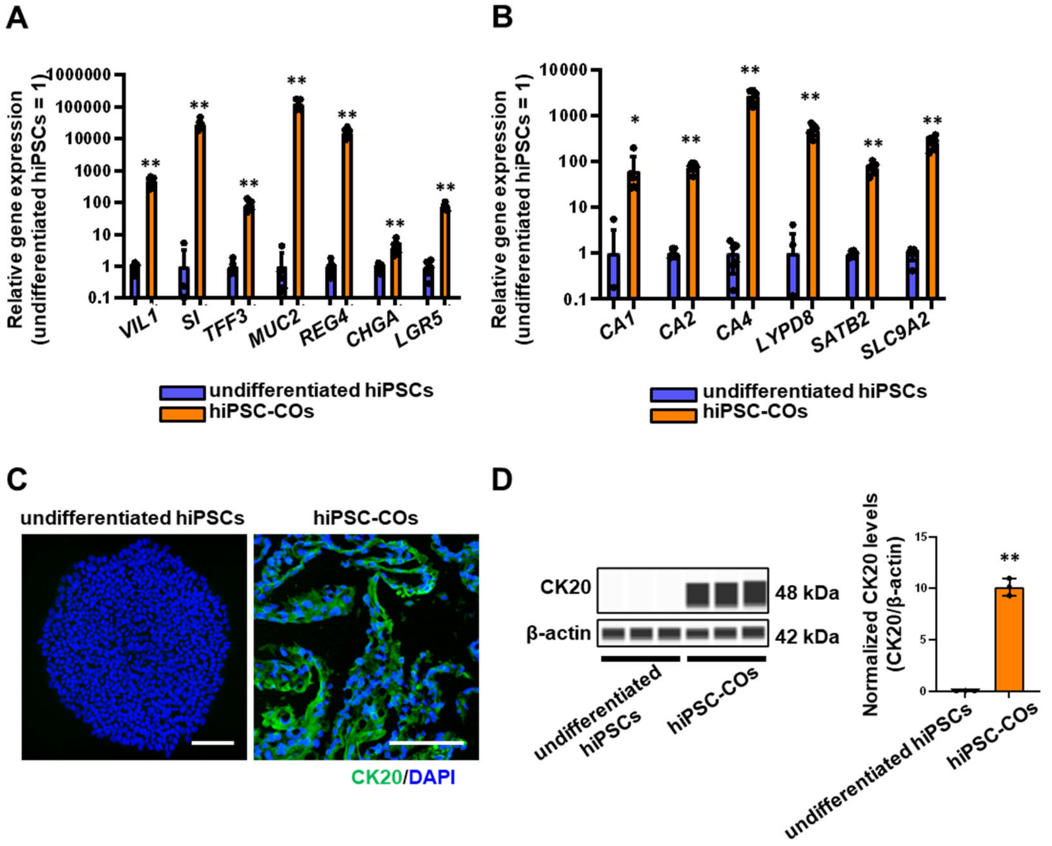

Figure S1. Generation of colon organoids from hiPSCs, Related to Figure 1

(**A**) Gene expression of intestinal epithelial markers (*VIL1*, *SI*, *TFF3*, *MUC2*, *REG4*, *CHGA*, and *LGR5*) was examined in undifferentiated hiPSCs and hiPSC-derived colon organoids (hiPSC-COs) by RT-qPCR. Gene expression in undifferentiated hiPSCs was taken as 1.0. Unpaired two-tailed Student's *t*-test (**p<0.01). Data are shown as means ± SD (n = 6). (**B**) Gene expression of colonic epithelial markers (*CA1*, *CA2*, *CA4*, *LYPD8*, *SATB2*, and *SLC9A2*) in undifferentiated hiPSCs and hiPSC-COs was examined by RT-qPCR. Gene expression in undifferentiated hiPSCs was taken as 1.0. Unpaired two-tailed Student's *t*-test (*p<0.05, **p<0.01). Data are shown as means ± SD (n = 6). (**C**) Immunofluorescence images of CK20 (green) in undifferentiated hiPSCs and hiPSC-COs are shown. Nuclei were counterstained with DAPI (blue). Scale bars represent 100 µm. (**D**) Protein levels of CK20 in undifferentiated hiPSCs and hiPSC-COs were examined by Jess analysis. Unpaired two-tailed Student's t-test (**p<0.01). Data are shown as means ± SD (n = 3).

Figure S2

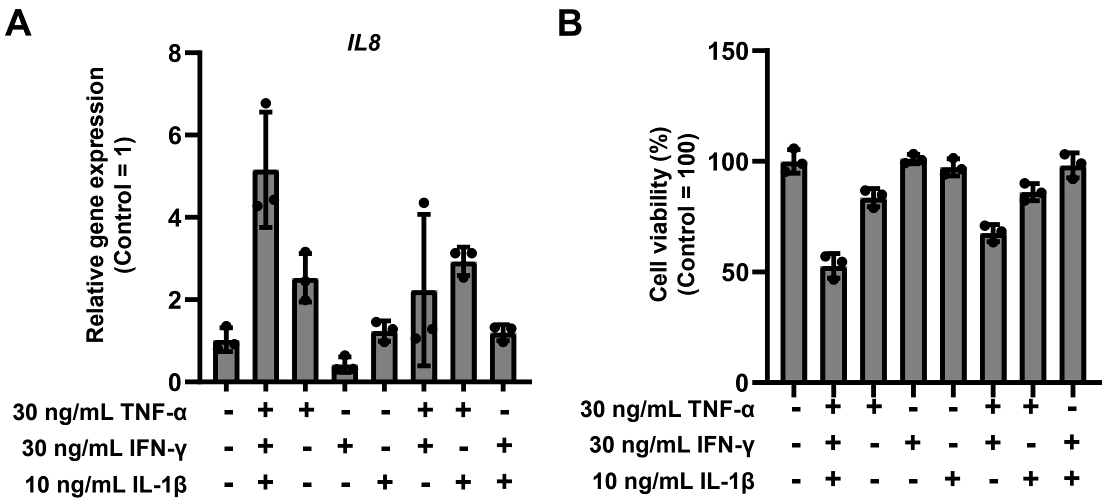

Figure S2. Cytokine combination screening in hiPSC-COs, Related to Figure 2

We screened for a combination of IBD-associated cytokines (TNF-α, IFN-γ, and IL-1β) to elicit an inflammatory response in hiPSC-COs. hiPSC-COs were treated with 30 ng/mL TNF-α, 30 ng/mL IFN-γ, or 10 ng/mL IL-1β for 8 days. (**A**) *IL8* gene expression in 30 ng/mL TNF-α-, 30 ng/mL IFN-γ-, or 10 ng/mL IL-1β-treated hiPSC-COs was examined by RT-qPCR. Gene expression in control hiPSC-COs was taken as 1.0. One-way ANOVA followed by the Tukey post-hoc test. *P*-values for all comparisons are shown in **Table S2**. Data are shown as means ± SD (n = 3). (**B**) The cell viability of 30 ng/mL TNF-α-, 30 ng/mL IFN-γ-, or 10 ng/mL IL-1β-treated hiPSC-COs was examined by WST-8 assay. One-way ANOVA followed by the Tukey post-hoc test. *P*-values for all comparisons are shown in **Table S3**. Data are shown as means ± SD (n = 3).

Figure S3

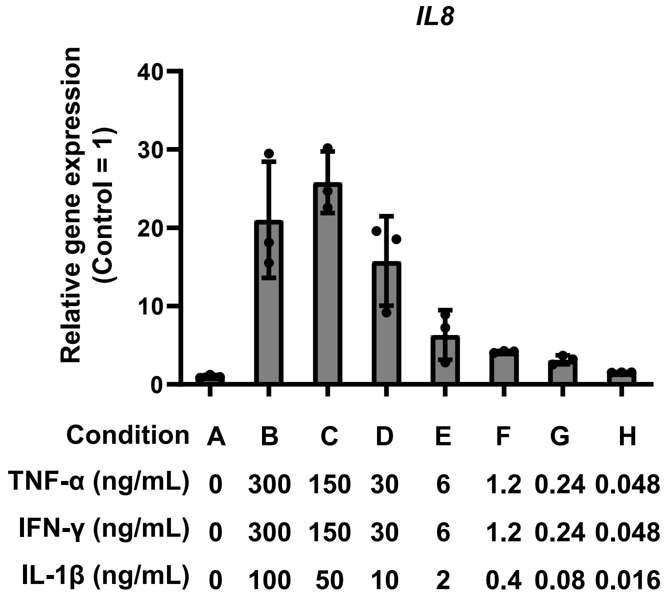

Figure S3. Dose optimization of TNF-α, IFN-γ, and IL-1β in hiPSC-COs, Related to Figure 2

hiPSC-COs were treated with different concentrations of TNF-α, IFN-γ, and IL-1β for 8 days. *IL8* gene expression in 3CK-treated hiPSC-COs was examined by RT-qPCR. Gene expression in control hiPSC-COs was taken as 1.0. One-way ANOVA followed by the Tukey post-hoc test. *P*-values for all comparisons are shown in **Table S4**. Data are shown as means ± SD (n = 3).

Figure S4

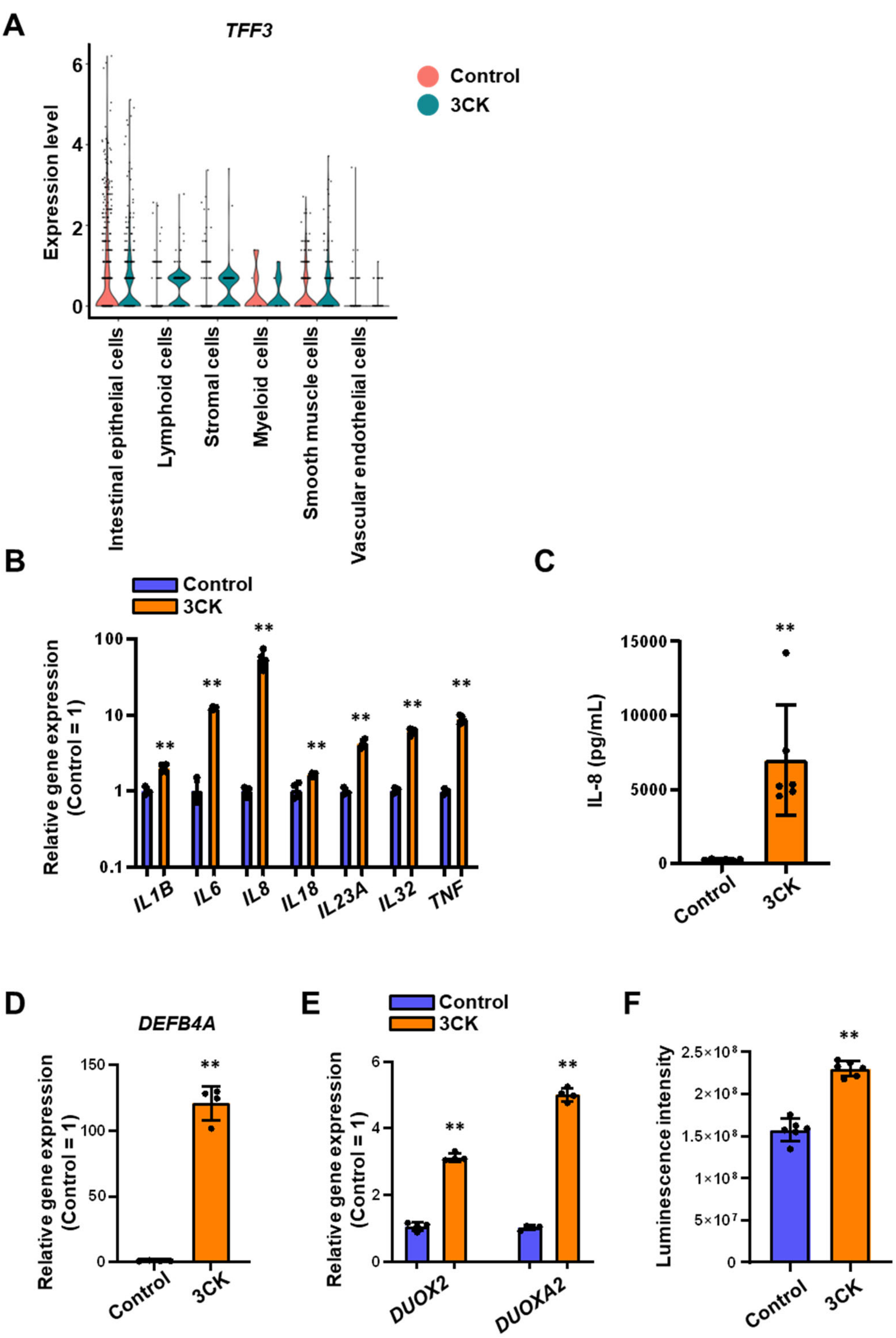

Figure S4. Inflammatory responses in 3CK-treated hiPSC-COs, Related to Figure 3
hiPSC-COs were treated with TNF-α (30 ng/mL), IFN-γ (30 ng/mL), and IL-1β (10 ng/mL) (referred to as 3CK) for 8 days. (**A**) scRNA-seq analysis of control and 3CK-

treated hiPSC-COs was performed. Violin plots show gene expression of *TFF3*, a goblet cell marker, in control and 3CK-treated hiPSC-COs. (**B**) Gene expression of cytokines (*IL1B*, *IL6*, *IL8*, *IL18*, *IL23A*, *IL32*, and *TNF*) in control and 3CK-treated hiPSC-COs was examined by RT-qPCR. Gene expression in control hiPSC-COs was taken as 1.0. Unpaired two-tailed Student's *t*-test (**$p<0.01$). Data are shown as means ± SD ($n = 4$). (**C**) Concentrations of IL-8 secreted by control and 3CK-treated hiPSC-COs were examined by ELISA. Unpaired two-tailed Student's *t*-test (**$p<0.01$). Data are shown as means ± SD ($n = 6$). (**D**) *DEFB4A* expression in control and 3CK-treated hiPSC-COs was examined by RT-qPCR. Gene expression in control hiPSC-COs was taken as 1.0. Unpaired two-tailed Student's *t*-test (**$p<0.01$). Data are shown as means ± SD ($n = 4$). (**E**) Gene expression of *DUOX2* and *DUOXA2* in control and 3CK-treated hiPSC-COs was examined by RT-qPCR. Gene expression in control hiPSC-COs was taken as 1.0. Unpaired two-tailed Student's *t*-test (**$p<0.01$). Data are shown as means ± SD ($n = 4$). (**F**) At 8 days after 3CK treatment, LDH released into the cell culture supernatant was measured. LDH release in control hiPSC-COs was taken as 1.0. Unpaired two-tailed Student's *t*-test (**$p<0.01$). Data are shown as means ± SD ($n = 6$).

Figure S5

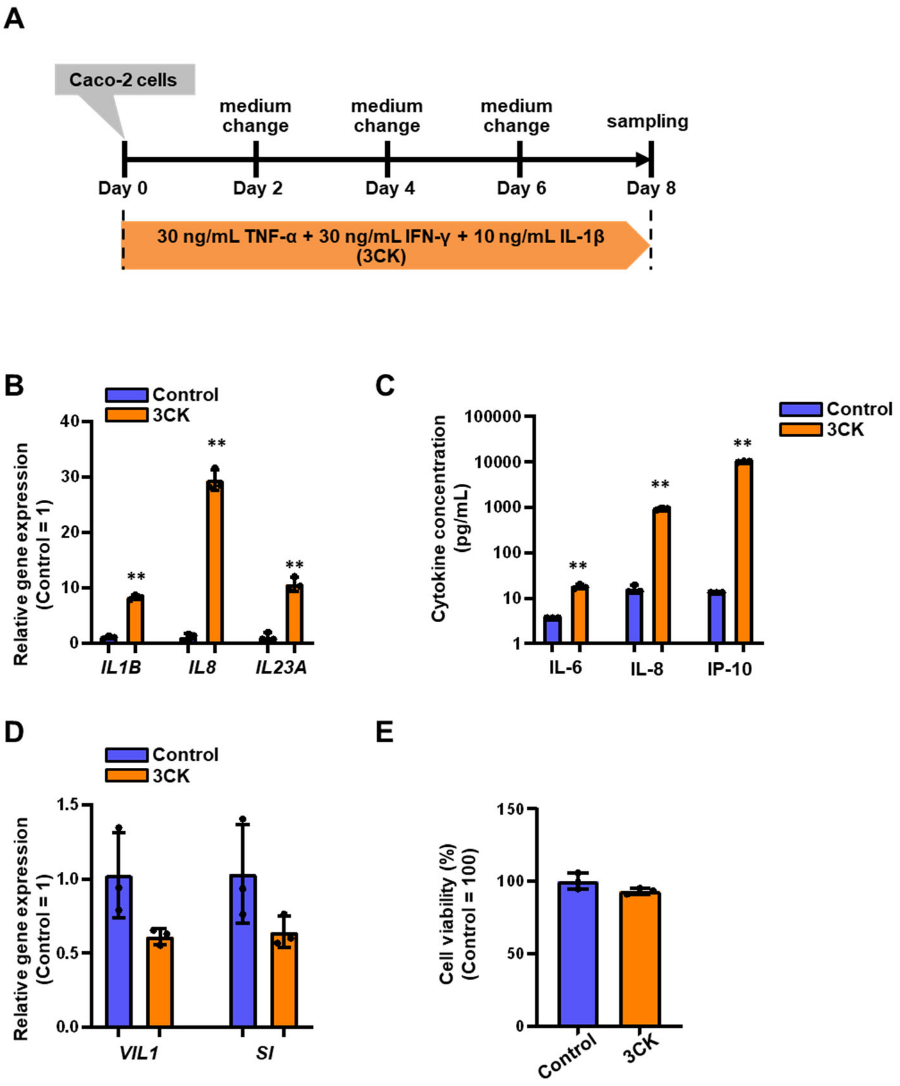

Figure S5. Characterization of 3CK-treated Caco-2 cells, Related to Figure 3

(**A**) Caco-2 cells were treated with TNF-α (30 ng/mL), IFN-γ (30 ng/mL), and IL-1β (10 ng/mL) (referred to as 3CK) for 8 days. (**B**) Gene expression of cytokines (*IL1B*, *IL8*, and *IL23A*) in control and 3CK-treated Caco-2 cells was examined by RT-qPCR. Gene expression in control hiPSC-COs was taken as 1.0. Unpaired two-tailed Student's *t*-test (**p<0.01). Data are shown as means ± SD (n = 3). (**C**) A bead-based multiplex immunoassay (LEGENDplex analysis) was performed to evaluate the secretion of cytokines (IL-6, IL-8, and IP-10 [CXCL10]) in the culture supernatants of control or 3CK-treated Caco-2 cells. Unpaired two-tailed Student's *t*-test (**p<0.01). Data are shown as means ± SD (n = 3). (**D**) Gene expression of *VIL1* and *SI* in control and 3CK-treated Caco-2 cells was examined by RT-qPCR. Gene expression in control Caco-2 was

taken as 1.0. Unpaired two-tailed Student's *t*-test. Data are shown as means ± SD ($n = 3$). (**E**) The cell viability of control and 3CK-treated Caco-2 cells was examined by WST-8 assay. Unpaired two-tailed Student's *t*-test. Data are shown as means ± SD ($n = 3$).

Figure S6

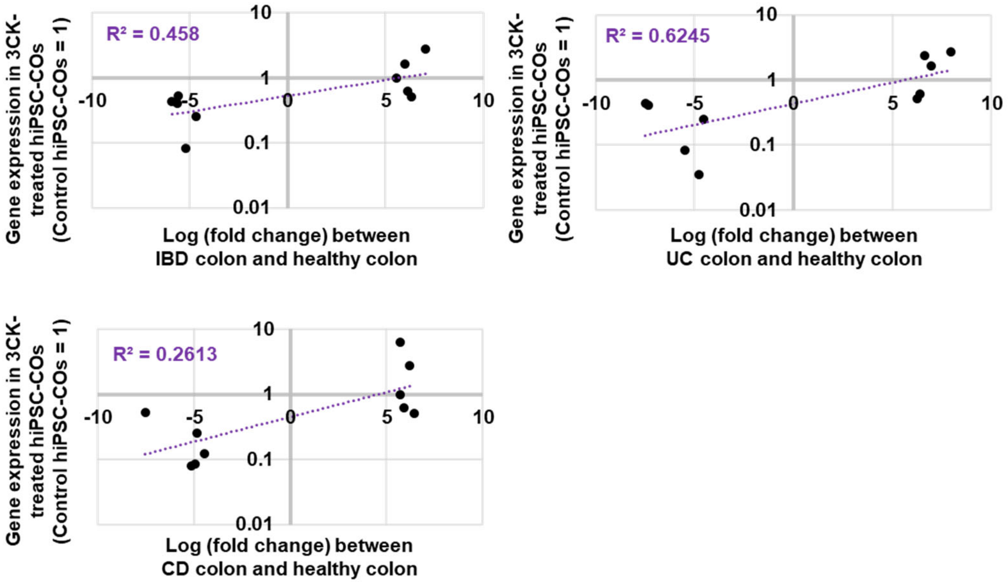

Figure S6. Comparison of 3CK-treated hiPSC-COs and UC patient colon, Related to Figure 5

hiPSC-COs were treated with TNF-α (30 ng/mL), IFN-γ (30 ng/mL), and IL-1β (10 ng/mL) for 8 days. Comparison of gene expression profiles among 3CK-treated hiPSC-COs, IBD colon, ulcerative colitis (UC) colon, and Crohn's disease (CD) colon. RNA-seq data obtained from the colon (active site) of IBD, UC, CD patients, and healthy individuals was used [1], and the 10 genes whose expression level was increased or decreased in the colon of IBD, UC, and CD patients compared to healthy colon were extracted (**Table S7**). The x-axis shows the log (fold change) between IBD, UC, or CD colon and healthy colon. The y-axis shows the fold change between control and 3CK-treated hiPSC-COs.

Figure S7

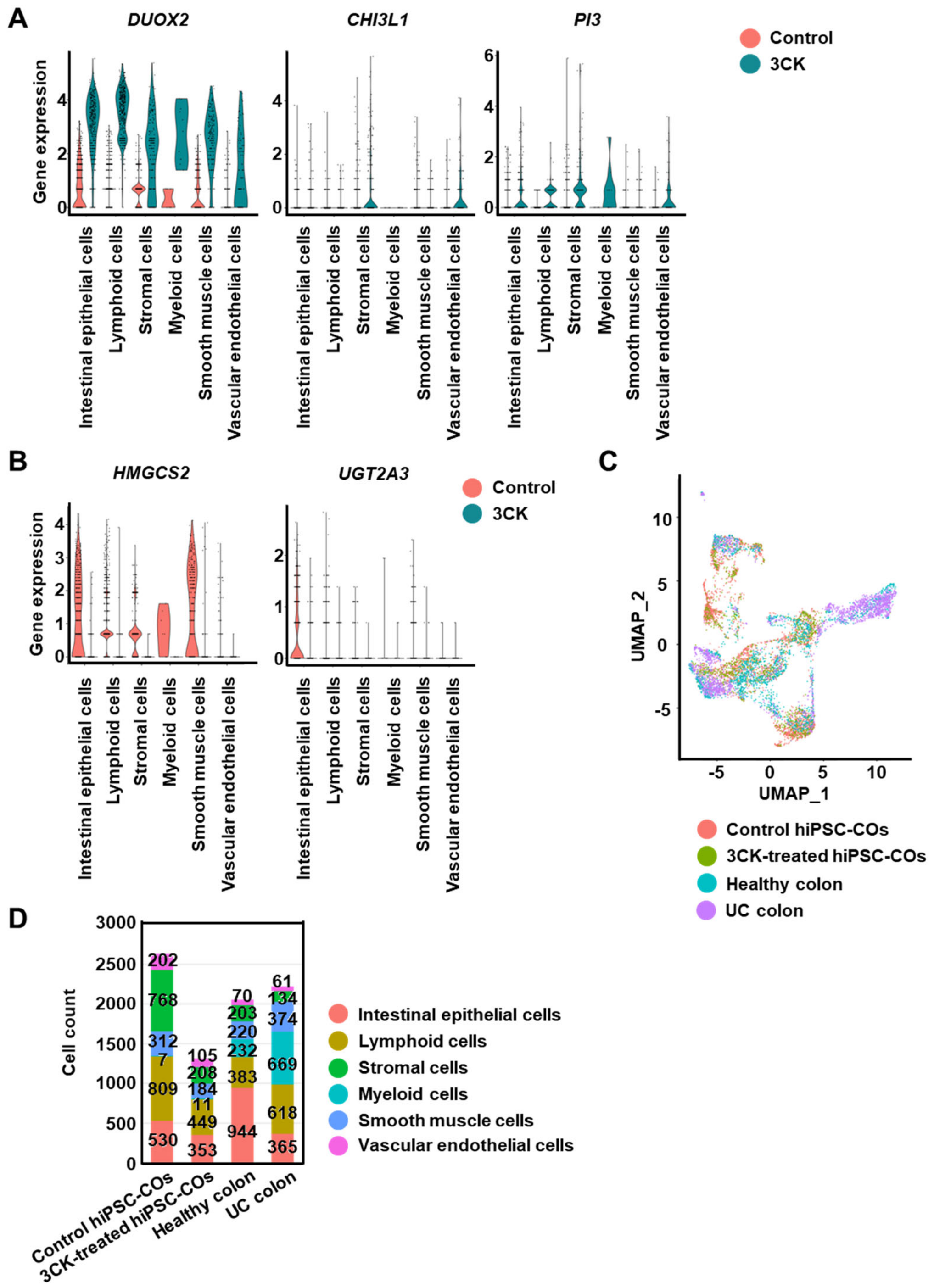

Figure S7. scRNA-seq analysis of 3CK-treated hiPSC-COs, Related to Figure 5

hiPSC-COs were treated with TNF-α (30 ng/mL), IFN-γ (30 ng/mL), and IL-1β (10 ng/mL) (referred to as 3CK) for 8 days. scRNA-seq analysis of control and 3CK-treated hiPSC-COs was performed. (**A, B**) Violin plots show the gene expression of *DUOX2*, *CHI3L1*, *PI3* (the upregulated genes in the UC colon compared to the healthy colon) (**A**)

and *HMGCS2*, *UGT2A3* (the downregulated gene in the UC colon compared to the healthy colon) (**B**) in control and 3CK-treated hiPSC-COs. (**C**) scRNA-seq analysis of control, 3CK-treated hiPSC-COs, healthy colon, and UC colon was performed. UMAP plots of control, 3CK-treated hiPSC-COs, healthy colon, and UC colon are shown. (**D**) The cell counts of each cluster of control, 3CK-treated hiPSC-COs, healthy colon, and UC colon are shown.

Figure S8

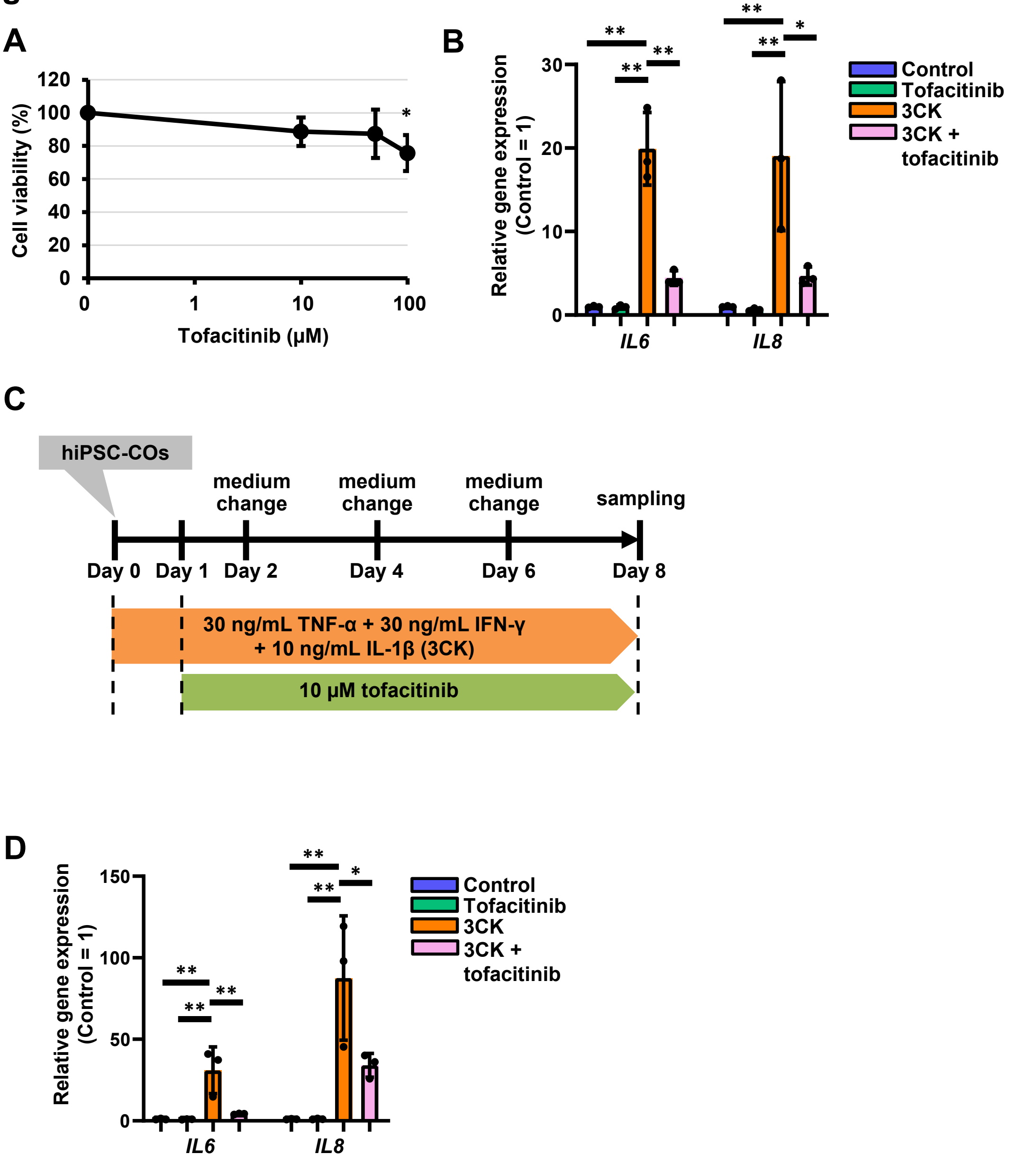

Figure S8. Evaluation of tofacitinib using 3CK-treated hiPSC-COs, Related to Figure 7

(**A**) The Cell viability of hiPSC-COs treated with various concentrations (0, 10, 50, and 100 μM) of tofacitinib was examined by WST-8 assay. Data are shown as means ± SD (n = 3). One-way ANOVA followed by Dunnett's post hoc test (*$p < 0.05$, compared with control hiPSC-COs). (**B**) Gene expression of cytokines (*IL6* and *IL8*) in control, tofacitinib-, 3CK-, or 3CK and tofacitinib-treated hiPSC-COs was examined by RT-qPCR. Gene expression in control hiPSC-COs was taken as 1.0. One-way ANOVA followed by the Tukey post-hoc test (*p<0.05, **p<0.01). Data are shown as means ± SD (n = 3). (**C**)

At 1 day after treatment with TNF-α (30 ng/mL), IFN-γ (30 ng/mL), and IL-1β (10 ng/mL), hiPSC-COs were treated with or without tofacitinib (10 μM). (**D**) Gene expression of cytokines (*IL6* and *IL8*) in control, tofacitinib-, 3CK-, or 3CK and tofacitinib-treated hiPSC-COs was examined by RT-qPCR. Gene expression in control hiPSC-COs was taken as 1.0. One-way ANOVA followed by the Tukey post-hoc test (*$p<0.05$, **$p<0.01$). Data are shown as means ± SD ($n = 3$).

Table S1. Gene list for cell-type annotation, Related to STAR Methods

Cluster	Genes
Intestinal epithelial cells	*UNC5CL, C1orf177, TRIM31, LINC00955, RP3-417L20.4, HMGN1P20, ALPI, RP11-395B7.2, EGFR-AS1, RP11-462G2.1, DEFB1, CA7, RP11-468N14.13, ARL14, AC024592.9*
Lymphoid cells	*IL17A, ICOS, SH2D1A, GZMK, RP11-553L6.2, TRGC1, RP11-104L21.3, TRDC, GZMA, VPREB1, CCR4, LTA, NCR2, CD247, RP11-94L15.2*
Stromal cells	*RP11-13P5.2, MSC, RP1-283K11.3, RP5-1077H22.1, CTD-2541J13.1, AC002511.2, AC002511.3, MMP27, CTD-2334D19.1, CXCL14, FZD10, PDGFRA, AC003090.1, RP1-288H2.4, ITGA11*
Myeloid cells	*VSIG4, RPL32P1, CD207, AP000439.1, HCAR3, C5orf20, AC010492.4, P2RY13, LILRB2, LILRB5, IL1B, C1QB, CD163, C1QC, DNASE1L3*
Smooth muscle cells	*ACTG2, GREM2, CNN1, RP11-467I20.6, WIF1, TACR2, OVCH2, FRMD6-AS2, DES, MYH11, RP11-626H12.1, ANO1, CHRDL2, TAGLN, FBXL22*
Vascular endothelial cells	*SOX17, GPIHBP1, PRND, CLEC3B, FLT1, MMRN2, BTNL9, APLNR, LHX6, NOTCH4, RP11-3K16.2, ESAM, RP11-328D5.1, ESM1, GJA5*

Table S2. *P*-values for all comparisons in Figure S2A

	P-value
Control vs TNF-α+IFN-γ+IL-1β	0.0005
Control vs TNF-α	0.4419
Control vs IFN-γ	0.9867
Control vs IL-1β	>0.9999
Control vs TNF-α+IFN-γ	0.6875
Control vs TNF-α+IL-1β	0.1963
Control vs IFN-γ+IL-1β	>0.9999
TNF-α+IFN-γ+IL-1β vs TNF-α	0.0318
TNF-α+IFN-γ+IL-1β vs IFN-γ	0.0001
TNF-α+IFN-γ+IL-1β vs IL-1β	0.0009
TNF-α+IFN-γ+IL-1β vs TNF-α+IFN-γ	0.0141
TNF-α+IFN-γ+IL-1β vs TNF-α+IL-1β	0.0909
TNF-α+IFN-γ+IL-1β vs IFN-γ+IL-1β	0.0008
TNF-α vs IFN-γ	0.1213
TNF-α vs IL-1β	0.6135
TNF-α vs TNF-α+IFN-γ	0.9998
TNF-α vs TNF-α+IL-1β	0.9989
TNF-α vs IFN-γ+IL-1β	0.5803
IFN-γ vs IL-1β	0.9358
IFN-γ vs TNF-α+IFN-γ	0.2428
IFN-γ vs TNF-α+IL-1β	0.0435
IFN-γ vs IFN-γ+IL-1β	0.9497
IL-1β vs TNF-α+IFN-γ	0.843
IL-1β vs TNF-α+IL-1β	0.3087
IL-1β vs IFN-γ+IL-1β	>0.9999
TNF-α+IFN-γ vs TNF-α+IL-1β	0.9701
TNF-α+IFN-γ vs IFN-γ+IL-1β	0.817
TNF-α+IL-1β vs IFN-γ+IL-1β	0.2846

Table S3. ***P*****-values for all comparisons in Figure S2B**

	P-value
Control vs TNF-α+IFN-γ+IL-1β	<0.0001
Control vs TNF-α	0.0061
Control vs IFN-γ	>0.9999
Control vs IL-1β	0.9939
Control vs TNF-α+IFN-γ	<0.0001
Control vs TNF-α+IL-1β	0.0233
Control vs IFN-γ+IL-1β	0.9994
TNF-α+IFN-γ+IL-1β vs TNF-α	<0.0001
TNF-α+IFN-γ+IL-1β vs IFN-γ	<0.0001
TNF-α+IFN-γ+IL-1β vs IL-1β	<0.0001
TNF-α+IFN-γ+IL-1β vs TNF-α+IFN-γ	0.0145
TNF-α+IFN-γ+IL-1β vs TNF-α+IL-1β	<0.0001
TNF-α+IFN-γ+IL-1β vs IFN-γ+IL-1β	<0.0001
TNF-α vs IFN-γ	0.0034
TNF-α vs IL1b	0.0259
TNF-α vs TNF-α+IFN-γ	0.0078
TNF-α vs TNF-α+IL-1β	0.9962
TNF-α vs IFN-γ+IL-1β	0.0167
IFN-γ vs IL-1β	0.9606
IFN-γ vs TNF-α+IFN-γ	<0.0001
IFN-γ vs TNF-α+IL-1β	0.0131
IFN-γ vs IFN-γ+IL-1β	0.9898
IL-1β vs TNF-α+IFN-γ	<0.0001
IL-1β vs TNF-α+IL-1β	0.0936
IL-1β vs IFN-γ+IL-1β	>0.9999
TNF-α+IFN-γ vs TNF-α+IL-1β	0.0021
TNF-α+IFN-γ vs IFN-γ+IL-1β	<0.0001
TNF-α+IL-1β vs IFN-γ+IL-1β	0.0619

Table S4. *P*-values for all comparisons in Figure S3

	P-value
Condition A vs Condition B	0.0002
Condition A vs Condition C	<0.0001
Condition A vs Condition D	0.0038
Condition A vs Condition E	0.674
Condition A vs Condition F	0.9631
Condition A vs Condition G	0.9959
Condition A vs Condition H	>0.9999
Condition B vs Condition C	0.7688
Condition B vs Condition D	0.6806
Condition B vs Condition E	0.0039
Condition B vs Condition F	0.001
Condition B vs Condition G	0.0006
Condition B vs Condition H	0.0002
Condition C vs Condition D	0.0719
Condition C vs Condition E	0.0002
Condition C vs Condition F	<0.0001
Condition C vs Condition G	<0.0001
Condition C vs Condition H	<0.0001
Condition D vs Condition E	0.1021
Condition D vs Condition F	0.028
Condition D vs Condition G	0.0148
Condition D vs Condition H	0.0053
Condition E vs Condition F	0.9959
Condition E vs Condition G	0.9633
Condition E vs Condition H	0.7703
Condition F vs Condition G	>0.9999
Condition F vs Condition H	0.9863
Condition G vs Condition H	0.9993

Table S5. The cell viability in each condition of Figure 2D

	Cell viability (%)
Condition A	100.0 ± 9.7
Condition B	19.6 ± 7.3
Condition C	19.3 ± 1.8
Condition D	45.3 ± 21.0
Condition E	65.5 ± 7.0
Condition F	73.4 ± 7.7
Condition G	98.4 ± 11.2
Condition H	115.1 ± 22.7

Table S6. *P*-values for all comparisons in Figure 2D

	P-value
Condition A vs Condition B	<0.0001
Condition A vs Condition C	<0.0001
Condition A vs Condition D	0.0018
Condition A vs Condition E	0.0725
Condition A vs Condition F	0.2565
Condition A vs Condition G	>0.9999
Condition A vs Condition H	0.8336
Condition B vs Condition C	>0.9999
Condition B vs Condition D	0.2907
Condition B vs Condition E	0.0092
Condition B vs Condition F	0.0021
Condition B vs Condition G	<0.0001
Condition B vs Condition H	<0.0001
Condition C vs Condition D	0.2824
Condition C vs Condition E	0.0088
Condition C vs Condition F	0.0021
Condition C vs Condition G	<0.0001
Condition C vs Condition H	<0.0001
Condition D vs Condition E	0.5617
Condition D vs Condition F	0.2048
Condition D vs Condition G	0.0024
Condition D vs Condition H	0.0001
Condition E vs Condition F	0.9938
Condition E vs Condition G	0.0949
Condition E vs Condition H	0.0047
Condition F vs Condition G	0.3192
Condition F vs Condition H	0.0201
Condition G vs Condition H	0.7586

Table S7. The percentage of apoptotic, necrotic, and live cells in hiPSC-COs treated with 3CK for 2 days, Related to Figure 3

	Control (%)	3CK (%)
Apoptotic cells	0.03 ± 0.04	0.03 ± 0.02
Necrotic cells	0.09 ± 0.08	0.03 ± 0.02
Live cells	99.85 ± 0.12	99.96 ± 0.06

Table S8. *P*-values for all comparisons in Figure 4B

	IL6	*IL8*	*CCL2*
	P-value	*P*-value	*P*-value
Vascular endothelial cells vs Stromal cells	0.9536	0.1353	0.6421
Vascular endothelial cells vs Smooth muscle cells	<0.0001	<0.0001	<0.0001
Vascular endothelial cells vs Myeloid cells	0.0036	0.3248	0.0139
Vascular endothelial cells vs Lymphoid cells	<0.0001	<0.0001	<0.0001
Vascular endothelial cells vs Intestinal epithelial cells	<0.0001	<0.0001	<0.0001
Stromal cells vs Smooth muscle cells	<0.0001	<0.0001	<0.0001
Stromal cells vs Myeloid cells	0.0005	0.9272	0.001
Stromal cells vs Lymphoid cells	<0.0001	<0.0001	<0.0001
Stromal cells vs Intestinal epithelial cells	<0.0001	<0.0001	<0.0001

Table S9. Gene list used in Figure S6

IBD genes	UC genes	CD genes
DUOX2	DUOX2	HLA-DPA1
HLA-DPA1	PI3	DUOX2
FN1	ABCA12	FN1
PI3	FN1	CD74
HLA-DPB1	HLA-DPA1	HLA-DPB1
SHISA9	SHISA9	ZNF385B
SLC6A4	CNTFR	SHISA9
FAM3B	SLC6A4	SLC6A4
SLC51A	SLC51A	KHDRBS2
SLC38A4	SLC38A4	FAM3B

Table S10. Primers used for RT-qPCR analysis, Related to STAR Methods

Gene	Forward (5'->3')	Reverse (5'->3')
GAPDH	GGAGCGAGATCCCTCCAAAAT	GGCTGTTGTCATACTTCTCATGG
VIL1	CTGAGCGCCCAAGTCAAAG	AGCAGTCACCATCGAAGAAGC
SI	TCCAGCTACTACTCGTGTGAC	CCCTCTGTTGGGAATTGTTCTG
TFF3	CCAAGCAAACAATCCAGAGCA	GCTCAGGACTCGCTTCATGG
MUC2	GAGGGCAGAACCCGAAACC	GGCGAAGTTGTAGTCGCAGAG
REG4	CTGCTCCTATTGCTGAGCTG	GGACTTGTGGTAAAACCATCCAG
CHGA	TAAAGGGGATACCGAGGTGATG	TCGGAGTGTCTCAAAACATTCC
LGR5	CTCCCAGGTCTGGTGTGTTG	GAGGTCTAGGTAGGAGGTGAAG
CA1	TTGAGGACAACGATAACCGATCA	CTACGTGAAGCTCGGCAGAAT
CA2	GGGTACGGCAAACACAACG	GGCTGTATGAGTGTCGATGTC
CA4	CTGGTGCTACGAGGTTCAAGC	GAAGAAGAAGCGTCCCAGTTT
LYPD8	CTGAAGAACGTGTCCAGCAA	CTGACAGGTGGCGTTACTGA
SATB2	GCAGTTGGACGGCTCTCTT	CACCTTCCCAGCTTGATTATTCC
SLC9A2	CAGATCCCCTTCGAGATCACC	GGGAGACTTCTCATCAACACCAA
DEFB4A	GGTGGTATAGGCGATCCTGTT	AGGGCAAAAGACTGGATGACA
DUOX2	CTGGGTCCATCGGGCAATC	GTCGGCGTAATTGGCTGGTA
DUOXA2	AACGGCGTACTGCCTTTTTAC	GAGAAGAACTCTCACCAACCAAA
IL1B	ATGATGGCTTATTACAGTGGCAA	GTCGGAGATTCGTAGCTGGA
IL6	ACTCACCTCTTCAGAACGAATTG	CCATCTTTGGAAGGTTCAGGTTG
IL8	TTTTGCCAAGGAGTGCTAAAGA	AACCCTCTGCACCCAGTTTTC
IL18	TCTTCATTGACCAAGGAAATCGG	TCCGGGGTGCATTATCTCTAC
IL23A	CTCAGGGACAACAGTCAGTTC	ACAGGGCTATCAGGGAGCA
IL32	TGGCGGCTTATTATGAGGAGC	CTCGGCACCGTAATCCATCTC
TNF	CCTCTCTCTAATCAGCCCTCTG	GAGGACCTGGGAGTAGATGAG
HLA-DPA1	ATGCGCCCTGAAGACAGAATG	ACACATGGTCCGCCTTGATG
FN1	CGGTGGCTGTCAGTCAAAG	AAACCTCGGCTTCCTCCATAA
PI3	CACGGGAGTTCCTGTTAAAGG	TCTTTCAAGCAGCGGTTAGGG
HLA-DPB1	CAGCACCACAACCTGCTTG	CCATTCAGGAACCATCGGACT
SHISA9	TCGGGCGACTTCATCTTCTG	TGATGTAGACGATCAGGTTGGT
SLC6A4	ATGGAGACGACGCCCTTGA	CTGTAGAACTCCGTTTTCCTGAC
FAM3B	ACACCTATGCCTACAGGTTACT	CAAAACATCGTGTTGCTGTCAC
SLC51A	CTGGGCTCCATTGCCATCTT	CACGGCATAAAACGAGGTGAT

SLC38A4	AGAAATTCCAAATACCCTGCCC	GAAGCGTGTTGTTGAATGACAG
ABCA12	TCTTTTCTTCGCAATGGTTCCT	GCTGGCATGACTTCTCTATCAAA
CNTFR	CTGGGCTCTGACGTGACAC	GTGGAAGCAGGCGTAGAGG
CD74	GATGACCAGCGCGACCTTATC	GTGACTGTCAGTTTGTCCAGC
ZNF385B	TTTCCAGTGGACAGTAGTTCTGC	ATAACCGCTTTTTGCACAGGA
KHDRBS2	CCCTCGGGGAAGCACTGTA	TGGGTCGCATAGCTGTTATCAT

Table S11. Antibodies used for immunofluorescence staining, Related to STAR Methods

Name	Catalog number	Host	Company
Cytokeratin 20 Polyclonal antibody	17329-1-AP	Rabbit	Proteintech
ZO-1 Antibody	5406	Rabbit	Cell Signaling
Donkey anti-Rabbit IgG (H+L) Highly Cross-Adsorbed Secondary Antibody, Alexa Fluor™ 488	A-21206	Donkey	Thermo Fisher Scientific
Goat anti-Rabbit IgG (H+L) Cross-Adsorbed Secondary Antibody, Alexa Fluor™ 594	A-11012	Goat	Thermo Fisher Scientific

Table S12. Antibodies used for Jess analysis, Related to STAR Methods

Name	Catalog number	Host	Company
Monoclonal anti-β-actin	A5441	Mouse	Sigma-Aldrich
Anti-villin antibody [EPR3490]	ab109516	Rabbit	Abcam
Cytokeratin 20 monoclonal antibody	60183-1-Ig	Mouse	Proteintech

Supplemental reference

1. Sæterstad, S., Østvik, A.E., Røyset, E.S., Bakke, I., Sandvik, A.K., and Granlund, A.V.B. (2022). Profound gene expression changes in the epithelial monolayer of active ulcerative colitis and Crohn's disease. PLoS One *17*, e0265189. 10.1371/journal.pone.0265189.
